# Plant–soil–microbiome interactions: mechanisms, advances, and challenges in sustainable agriculture and healthy agroecosystems

**DOI:** 10.3389/fmicb.2026.1762743

**Published:** 2026-02-20

**Authors:** Jacek Panek, Agata Gryta, Wiktoria Maj, Mateusz Mącik, Karolina Oszust, Giorgia Pertile, Michał Pylak, Dominika Siegieda, Moritz Hallama, Ryusuke Hatano, Ellen Kandeler, Shamina Imran Pathan, Giacomo Pietramellara, Eligio Malusa, Jerzy Weber, Katarzyna Turnau, Sylwia Różalska, Magdalena Frąc

**Affiliations:** 1 Institute of Agrophysics, Polish Academy of Sciences, Lublin, Poland; 2 Soil Biology Department, Institute of Soil Science and Land Evaluation, University of Hohenheim, Stuttgart, Germany; 3 Research Faculty of Agriculture, Hokkaido University, Sapporo, Japan; 4 Department of Agriculture, Food, Environment and Forestry, University of Florence, Florence, Italy; 5 The National Institute of Horticultural Research, Skierniewice, Poland; 6 Wroclaw University of Environmental and Life Sciences, Wrocław, Poland; 7 Institute of Environmental Sciences, Jagiellonian University in Kraków, Kraków, Poland; 8 Department of Industrial Microbiology and Biotechnology, University of Lodz, Lodz, Poland

**Keywords:** biodiversity, climate change, microbiomes, plant resilience, regenerative agriculture, soil functionality, soil health

## Abstract

The focus of this article is to summarize current knowledge of plant-associated microbiomes, which play a key role in plant health and in maintaining soil quality. Such microbiomes, comprising bacteria, fungi, archaea, algae, nematodes, and protists, perform various functions, including nutrient transformation, pathogen protection, and stress mitigation. Microbial communities are commonly used as an indicator of ecosystem health. Soil microbiome diversity depends on environmental factors (including biotic and abiotic stresses), which can alter microbial composition, thereby modifying microbial interactions and plant resilience. Biofertilizers, biopreparations, and microbial inoculants or consortia have been utilized in agriculture to enhance soil properties, such as microbial diversity and enzymatic activity, and to prime plant immune responses, thereby promoting plant growth and health. Biofertilizers can significantly help plants adapt to environmental stresses and climate change, mitigating drought stress and reducing greenhouse gas emissions. Recent advances in DNA sequencing technologies, the computing power available to scientists, and the development of bioinformatics tools have made microbial community studies widely accessible. These tools enable the research and modeling of changes in the soil microbiome, plant disease susceptibility, and soil health. Multi-omics approaches to microbiomes are key to characterizing the microbiome and predicting plant diseases. Future research should focus primarily on understanding the interactions among soil, plants, and microbiomes. This approach will help develop climate-resilient plants and improve the health and functionality of agroecosystems. Key efforts closely aligned with the European Union’s goals and biodiversity strategies for sustainable agriculture and soil health restoration, as presented in this review, include studying the structures and functions of soil microbiomes, developing new assays, and designing and investigating microbial consortia to restore healthy communities. These strategies address contemporary challenges in agriculture, including vertical and urban farming and superfood production.

## Graphical abstract

**Figure:**
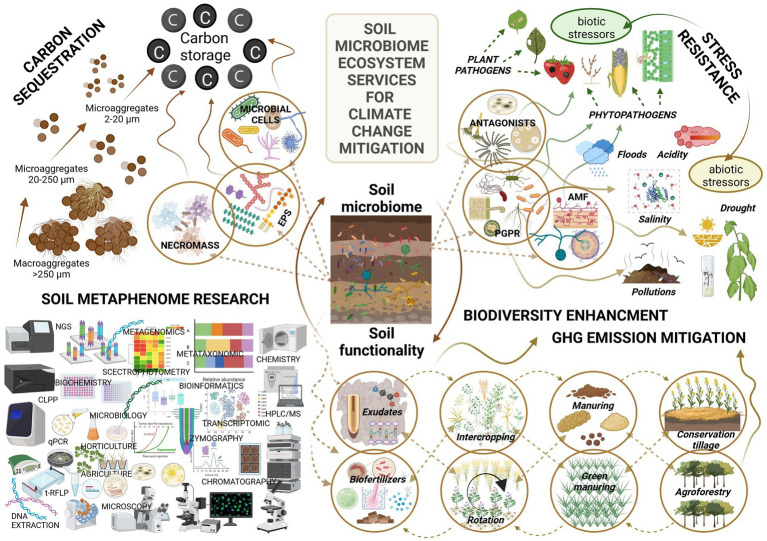

## Highlights

- Plant-associated microbiomes are crucial in maintaining plant health and soil quality.
- Soil microbiome diversity is shifted by environmental factors, impacting plant resilience.
- Biofertilizers and microbial inoculants can enhance soil quality, plant growth and health.
- Advances in DNA sequencing and bioinformatics, along with machine learning techniques, are essential for predicting soil microbiome changes.
- Soil-plant-microbiome interactions are essential to developing climate-resilient soils and plants.
- Innovative agricultural approaches are critical for the superfoods’ production, to ensure sustainable farming practices and food security.

## 1 Introduction

Current problems in sustainable agriculture include the threat of biodiversity loss, a significant issue in recent years. The threats most often mentioned include climate change, erosion, depletion of soil organic matter, agricultural intensification, and land-use changes. Therefore, the search for sustainable plant cultivation strategies is essential for maintaining the quality of the farming environment. These strategies are used to develop biotechnological solutions for sustainable and organic agriculture. The new vision of agriculture emphasizes the close connection between crop management and the soil and plant microbiome. Therefore, plant and soil microbiome management is increasingly used to: enhance the resistance of specific crops to pests, pathogens, drought or nutrients, select/develop pest control practices that are best in the context of sustainable food production, fully integrate biological agents/changes or microbiome control with crop management depending on the location, conditions and environmental factors (e.g., climate zone, soil type, biotic and abiotic stresses), develop biopreparations and methods for detecting phytopathogens for sustainable crop production management.

Therefore, the interactions between plants and microbiomes and their hosts are essential functional contexts. Plant–microbiome interactions have co-evolved to maintain the plant’s overall stability, functionality, and fitness as a holobiont (Trivedi et al., 2020). A plant holobiont is defined as an ecological and functional unit composed of the plant host and its associated microbiota, including bacteria, fungi, archaea, protists, viruses, and other microorganisms inhabiting both above- and belowground plant compartments (Mesny et al., 2023; Vandenkoornhuyse et al., 2015). These microbial partners interact with the host through metabolic, signaling, and immune-mediated processes, collectively shaping plant development, health, stress resilience, and adaptive capacity within a given environment (Berg et al., 2016; Berg et al., 2020). Plants are recognized as metaorganisms because the plant-associated microbiome comprises all microorganisms colonizing plant surfaces and internal tissues, including the rhizosphere, phyllosphere, endosphere, and spermosphere (Berg et al., 2017). These microorganisms can exert beneficial, neutral, or detrimental effects on the host and play key roles in nutrient cycling, pathogen suppression, modulation of plant immunity, and responses to biotic and abiotic stresses (Frąc et al., 2018; Jansson and Hofmockel, 2020; Siegieda et al., 2023; Trivedi et al., 2020).

**Figure 1:**
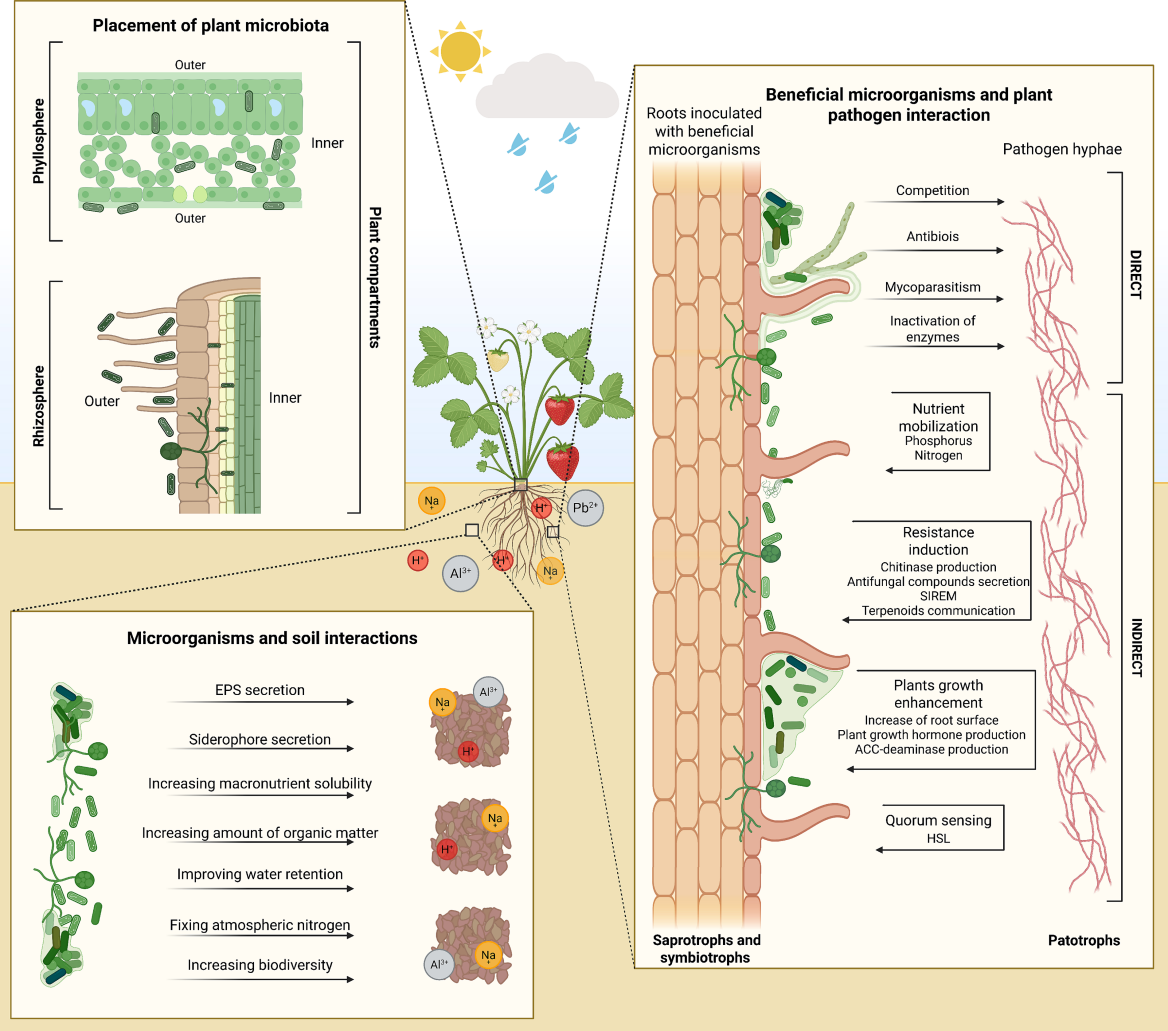
The interactions among plant compartments highlight how microorganisms contribute to soil health and enhance host plant growth. EPS – extracellular polymeric substances; SIREM – systemically induced root exudation of metabolites; HSL, homoserine lactone. Created with BioRender.com.

A plant microbiome comprises beneficial, neutral, and pathogenic microorganisms. The benefits of microorganisms to their host plants can be direct – including transformation and translocation of relevant nutrients in the soil to make them available to plants, protection against plant pathogens through antibiosis competition, production of hydrolytic enzymes, and mitigation of environmental stresses (Trivedi et al., 2016a). Benefits can also be indirect, as they enhance plant resistance responses (Pieterse et al., 2014). As illustrated in Figure 1, the complex interactions among plant compartments demonstrate how microorganisms actively contribute to soil health and promote host plant growth. Through mechanisms such as the production of extracellular polymeric substances (EPS), systemically induced root exudation of metabolites (SIREM), and signaling via homoserine lactones (HSL), microbial communities facilitate nutrient cycling, stress resilience, and plant–soil communication. These interconnected processes emphasize the functional integration of the soil and plant microbiome in maintaining ecosystem stability and productivity.

However, diseases are characterized by microbial dysbiosis and subsequent responses from specific microbes that can act as antagonists or synergists toward pathogens (Trivedi et al., 2020). A healthy microbiome, also known as the eubiosis state, can be defined by high microbial richness, maintenance of its symbiotic functions for the host, and resistance to changes under physiological stress (Kuntz et al., 2015). The plant-associated microbiome is often described in terms of trophic modes and ecological guilds, which are defined by specific attributes of individual microorganisms. Examples include pathotrophs, which obtain nutrients by harming host cells, and phagotrophs; symbiotrophs, which obtain nutrients by exchanging resources with host cells; and saprotrophs, which obtain nutrients by breaking down dead host cells (Oszust and Frąc, 2021).

Despite significant progress in microbiome research, major knowledge gaps persist in understanding the functional mechanisms linking microbial community composition with plant performance and soil health. Most studies focus on taxonomic profiling using high-throughput sequencing but lack insight into metabolic functions, signaling pathways, and interspecies interactions that drive ecosystem stability (Bender et al., 2016; Hartmann and Widmer, 2006). Furthermore, the temporal dynamics of microbiomes – how communities change across plant growth stages, seasons, and environmental stress events – remain largely uncharacterized (Shade et al., 2012). Similarly, the context-dependence of microbial functions under different soil types, climates, and management systems has not been systematically addressed, limiting the translation of laboratory findings into field-scale applications (Raaijmakers and Mazzola, 2016; Fierer, 2017).

Several unresolved questions hinder our ability to harness microbiomes for sustainable agriculture. These include:

- What governs the assembly and resilience of the plant–soil microbiome under environmental stress (Trivedi et al., 2020; Walters et al., 2018)?
- How do specific keystone taxa or microbial consortia mediate plant stress responses (Banerjee et al., 2018; Agler et al., 2016)?
- To what extent can microbiome manipulation (e.g., inoculants, biofertilizers) produce consistent outcomes across different agroecosystems (Berg et al., 2020)?
- What are the thresholds of microbial diversity necessary to maintain ecosystem function and resistance to pathogen invasion (Loreau et al., 2001)?

Understanding these questions is vital to predicting how soil microbial communities will respond to global change drivers such as warming, drought, and nutrient limitation (Cavicchioli et al., 2019).

Current approaches to studying the plant–soil microbiome are constrained by methodological and technological limitations. Although next-generation sequencing provides massive amounts of genetic data, it often fails to capture functional expression and metabolic activity *in situ* (Prosser, 2015; Jansson and Hofmockel, 2020). Culture-dependent methods still recover only a small fraction of microbial diversity, while metagenomic and metatranscriptomic techniques require complex bioinformatics pipelines and standardized protocols for data comparability (Franzosa et al., 2015; Knight et al., 2018). Additionally, short-term experimental designs and the lack of integrated multi-omics frameworks restrict our capacity to connect microbial genes to ecological outcomes (Bahram et al., 2018; van der Heijden and Hartmann, 2016). Addressing these limitations requires interdisciplinary strategies that combine long-term monitoring, experimental ecology, and predictive modeling to bridge the gap between descriptive and functional microbiome science (Berg et al., 2020; Trivedi et al., 2020).

Such topics as an exploration of interactions within the plant–soil-microbiome structure, their resilience to pathogens, studying their composition and employing advanced bioinformatic tools to find connections and impact of biodiversity shifts toward crop health and productivity, and ways to restore and induce healthy microbiome although were briefly discussed in the past, are not yet presented in condensed, linked together manner, what is the primary focus of this review.

This review examines the interactions between plants, soil, and their microbial communities, focusing on how influencing microbial composition can enhance agricultural sustainability and preserve or restore biodiversity.

## 2 Relationships between plant compartments and microbial benefits to soil and host plants

### 2.1 Composition and roles of the plant microbiome

Bacteria are the most dominant members of the plant microbiome and, to date, have been studied more intensively than other community members. Nevertheless, other organisms, including fungi, archaea, algae, nematodes, and protists, also play essential roles in plant functioning and health (Leach et al., 2017).

Collectively, these microorganisms form an integrated system that supports plant growth, nutrient cycling, and defense mechanisms within various plant compartments.

The subset of microorganisms commonly found within the plant microbiome and providing essential functions within the habitat is called the core microbiome (Trivedi et al., 2020), which is is directly associated with plant health (Banerjee et al., 2018). Microbial taxa that occupy central positions in microbial networks are referred to as *hub microorganisms* or *keystone species*. They maintain community structure and function regardless of their abundance (Trivedi et al., 2020). Host genomic regions involved in carbohydrate metabolism and stress responses influence the abundance of these hub microorganisms (Roman-Reyna et al., 2019), underscoring the tight connection between plant genetics and microbial composition. Hub microorganisms exert regulatory control over the network of microbial interactions, as their removal results in the loss of interactions (Trivedi et al., 2020).

### 2.2 Spatial structure: microbiomes across plant compartments

Biotic and abiotic factors shape the structure of the plant microbiome following ecological rules (Berg et al., 2016). Distinct microbial communities inhabit outer compartments (rhizosphere, phyllosphere, and sporosphere) and inner ones (endorhiza, endosphere, and spermosphere). This spatial organization reflects selective pressures from environmental conditions that shape plant compartments both below and above ground, ultimately influencing plant traits and functions (Ravanbakhsh et al., 2019).

Microbiome-mediated benefits often originate belowground, especially in the rhizosphere, and can be transmitted to aboveground parts via plant-mediated transport or signaling (van der Heijden and Hartmann, 2016). The seed microbiome plays a critical role by ensuring the vertical transmission of a co-evolved microbial community to the next generation (Berg et al., 2017).

### 2.3 Soil–plant connectivity and environmental interactions

Although individual plant compartments harbor distinct microbiomes, they remain functionally interconnected with the surrounding soil environment (Berg et al., 2017). Microorganisms modulate plant responses to environmental stress conditions, such as drought, salinity, and temperature fluctuations, through three primary pathways: (1) altering the fitness of individual plant genotypes; (2) modifying expression of plant traits related to stress tolerance, and (3) influencing natural selection pressures within plant populations (Trivedi et al., 2020). In this way, soil microbes do not merely react to environmental stress but actively shape how plants adapt to a changing climate.

Plants also tend to select stress-resilient microbial consortia under abiotic and biotic stress conditions (Fitzpatrick et al., 2019), reinforcing a feedback loop that links soil microbial composition to plant growth.

### 2.4 Microbial genes and functional interactions

Several microbial genes in plant holobionts mediate cooperative or competitive interactions among microbiome members (Hassani et al., 2018). For example, many bacteria encode antimicrobial mechanisms that regulate community diversity, distribution, and abundances of within the plant host (Blair et al., 2018). Traits related to pathogen suppression, such as genes for antifungal compound synthesis or protein secretion systems, are more likely to be found in the rhizosphere of disease-resistant plant varieties (Santos and Olivares, 2021). In contrast, the pathogen-induced activation of chitinase genes and biosynthetic clusters encoding non-ribosomal peptide synthetases (NRPSs) and polyketide synthases (PKSs) in endophytic root microbiomes supports disease suppression (Mandels and Andreotti, 1978).

Phyllosphere bacteria also possess diverse biosynthetic gene clusters (BGCs), including ribosomally synthesized peptides and terpene systems, which facilitate intermicrobial and interkingdom signaling (Muñoz et al., 2021). The detection of antibiotic resistance genes in plant further demonstrates microbes’ role in shaping microbial community dynamics (Larsson and Flach, 2021).

### 2.5 Communication and chemical signaling

Quorum sensing enables bacteria to coordinate collective behaviors through signaling molecules such as homoserine lactone (HSL) (Acet et al., 2021). These signals facilitate cooperation or competition among bacterial taxa and even influence interkingdom interactions-plants can perceive HSLs, leading to altered metabolism, immune activation, and root development (Fan et al., 2022). Different bacterial taxa can produce the same types of signaling molecules, allowing for cooperation or interference with other taxa (West et al., 2021). As signal concentrations accumulate in the soil environment, population-wide shifts in gene expression occur, affecting microbial metabolism and plant-microbe interactions (Lipa et al., 2017). However, few studies have mapped these metabolic and signaling interactions between soil and plant microbial communities. It is important to note that much metataxonomic and metagenomic research focuses solely on cataloging microbial species across different ecosystems and compartments. Moving beyond species cataloging, future research must focus on microbiome functionality – the biochemical processes driving plant–soil symbiosis (Frąc et al., 2018; Jansson and Baker, 2016).

### 2.6 Chemical and metabolic exchanges between soil and plant

Chemical compounds involved in inter- and intra-kingdom communication, called chemical mediators, such as terpenoids, serve dual roles in defense against herbivores and pathogens, as well as in microbial communication (Rosenkranz et al., 2021; Schmidt and Saha, 2021). Specific microbial colonization can trigger Systemically Induced Root Exudation of Metabolites (SIREM), facilitating long-distance communication across unconnected microhabitats in the rhizosphere (Zancarini et al., 2021). These dynamic exchanges demonstrate that soil microorganisms are integral not only to nutrient cycling but also to chemical signaling networks that maintain plant health.

### 2.7 Eco-evolutionary processes in microbiome assembly

Recent research emphasizes the role of eco-evolutionary mechanisms – including dispersal, selection, ecological drift, and diversification in shaping the plant microbiome (Cordovez et al., 2019). These forces determine microbial community composition at soil–plant interfaces, thereby influencing plant resilience under environmental stress.

Understanding these processes provides a predictive framework for screening beneficial microorganisms that enhance plant fitness or act as biocontrol agents under changing climatic conditions (Trivedi et al., 2020).

### 2.8 Toward soil microbiome management for climate resilience

Given the multiple functions of soil microbes, managing the rhizosphere microbiome offers a promising strategy to mitigate the impacts of climate change. Rhizosphere microorganisms can improve soil water retention through the production of extracellular polymeric substances (EPS), serve as carbon sinks by incorporating plant-derived carbon into stable forms, and enhance nutrient uptake via mycorrhizal associations or production of growth-promoting hormones such as indole-3-acetic acid (IAA) (Jansson and Hofmockel, 2020).

Thus, sustainable management of the soil microbiome not only benefits plant productivity but also contributes to global carbon cycling and ecosystem stability.

Finally, microbial diversity remains the cornerstone of plant health and biocontrol potential (Berg et al., 2016); however, defining what constitutes a “healthy microbiome” continues to challenge researchers worldwide (Trivedi et al., 2020).

Continuous study of soil–plant–microbe interactions will be critical for developing resilient agroecosystems in the face of global environmental change.

### 2.9 Integrative perspective: soil as the core regulator of plant health

The plant microbiome represents a complex web of independent organisms whose interactions are deeply rooted in soil. Since many microorganisms migrate from bulk soil into plant-associated zones, such as the rhizosphere (Gu et al., 2023; Siegieda et al., 2023), the soil microbiome functions as both the foundation and regulator of the entire plant holobiont. Maintaining soil microbial balance is therefore essential to sustain plant health, productivity, and ecosystem resilience.

## 3 Managing the soil and plant microbiome

### 3.1 Global patterns, ecological drivers, and knowledge gaps in soil microbiome diversity

Global patterns in the diversity and abundance of soil microbiomes depend on environmental factors and soil microbial biogeography, which are crucial for predicting ecosystem functions across a changing world (Chu et al., 2020). Today, knowledge concerning the ecological drivers of microbial community composition and biodiversity across different ecosystem types, such as soils (Tripathi et al., 2018), oceans (Hellweger et al., 2014), or freshwater (Filker et al., 2016). However, knowledge of the relationships between microbiomes and plant–soil-microbiome interactions remains limited (Chu et al., 2020). The capacity to predict changes in microbially driven functions remains limited, and research focusing on culturing and whole-genome sequencing is necessary to bridge these knowledge gaps. Understanding the distributions of soil microbial communities, from local to global scales, has substantially improved over the last two decades. However, in soil ecosystems, most studies have not identified the expected global-scale trend in soil biodiversity. The typical trend of decreasing diversity from the tropics to the poles was partially confirmed as a reduction in soil bacterial diversity from the equator to Antarctica (Delgado-Baquerizo et al., 2016). Bahram et al. (2018) reported that bacterial and fungal diversity exhibited opposite patterns across the latitudinal gradient in global topsoils: bacterial taxonomic diversity was highest in temperate habitats, whereas this pattern was not observed for fungi. Similarly to plant diversity, few studies have reported a decline in soil microbial diversity with increasing elevation (Delgado-Baquerizo et al., 2016). The results of Fierer and Jackson (2006) highlighted the relevance of soil pH as a fundamental driver of the bacterial diversity distribution and community composition across contrasting biomes. Regarding fungal communities, Tedersoo et al. (2014) found that climate is a significant ecological driver. The correlation networks used in microbial ecology (Barberán et al., 2012) improved our ability to quantify microbial co-occurrence patterns. However, the field of microbial networks is relatively new. It should be developed based on years of experience researching plant and animal communities (Thebault and Fontaine, 2010). However, it was recently discovered to play a crucial role in determining crop health (Siegieda et al., 2023). Although efforts have been made to predict future distributions of soil microbial communities, there remains a lack of ability to predict global soil biodiversity and ecosystem functions. There is a need to study plant and soil microbiomes to deepen our understanding of soil microbiome compositions and the temporal distribution of microbial communities, which are still largely unknown, to utilize microbes like engineers to support human development. One way to ensure such ideas is to use microbial-based solutions in agriculture.

#### 3.1.1 Impact of biofertilizers and bio-inoculants on soil microbiome

Research on the influence of biofertilizers and microbial inoculants on the status of the soil bacterial microbiome and mycobiome has emerged as an integral part of scientific work in the development of sustainable and organic agriculture (Mitter et al., 2021; Mahmud et al., 2021; Sudheer et al., 2020). Microbially-based solutions include both bioproducts containing microbial inoculants (Coleman-Derr and Tringe, 2014; Mącik et al., 2020a; Pylak et al., 2021) and new-era solutions based on soil–plant–microbiome interactions meaningful to plant breeding strategies (Berg et al., 2016). This is especially urgent under changing climate conditions, as the combined effects of soil and plants, considered as a holobiont, can yield unique traits and functions, including increased resistance to abiotic and biotic stressors (Berg et al., 2020).

One perspective is that the welfare of soil microorganisms is inextricably linked to maintaining high soil quality and fertility (Hartman et al., 2018; Kumar et al., 2021). Some researchers suggest that using strains of beneficial bacteria and fungi as biofertilizers reduces mineral fertilizer inputs and improves soil microbiological properties. These include enzymatic activity, the occurrence of beneficial microbial taxa, and the number of operational taxonomic units associated with metabolic processes (Alori and Babalola, 2018; Mącik et al., 2022), although there are also problems with this approach (Raymond et al., 2021).

High diversity among soil microorganisms is crucial for the proper functioning of the soil ecosystem, as the greater the number of different microbial strains, the greater the variety of their activities (Maron et al., 2018). It has been documented that applying biofertilizers can enhance soil microbial diversity. Wang G. et al. (2021) observed an increase in Chao1, ACE, and Shannon indices in soil inoculated with both *B. subtilis* and *B. amyloliquefaciens*, while Chen et al. (2021) found that *Trichoderma* agent and *B. subtilis* improved bacterial richness and diversity. Trabelsi et al. (2012), Kandasamy et al. (2019), and Mącik et al. (2020a) observed more terminal restriction fragments (T-RFs) in soils amended with biofertilizers, suggesting increased soil microbial biodiversity. Despite growing interest in biofertilizers as sustainable alternatives to chemical inputs, numerous studies have reported inconsistent, context-dependent effects across soil types, climatic zones, and crop systems. The variability in biofertilizer performance is often attributed to complex interactions between microbial inoculants and native soil microbiota, as well as to soil physicochemical properties, nutrient availability, and host plant genotype (Bashan et al., 2014; Timmusk et al., 2017). For instance, certain plant growth-promoting rhizobacteria (PGPR) show strong yield improvements in nutrient-poor or degraded soils but limited or no effects in fertile, microbially rich environments (Shaaban et al., 2025; Rouphael et al., 2015). Climatic conditions-particularly temperature, moisture, and pH-also influence inoculant establishment and persistence, leading to variable responses under field conditions (Vessey, 2003; Trivedi et al., 2020). Moreover, biofertilizers that enhance productivity in one crop species or region may fail or even negatively affect others due to differences in root exudate chemistry and microbiome compatibility (Bhattacharyya and Jha, 2012; Compant et al., 2019). These contradictions highlight the urgent need for context-specific formulation and precision microbiome management, integrating soil, crop, and environmental data to improve the predictability and reliability of biofertilizer applications.

#### 3.1.2 Potential of biocontrol agents in maintaining the health of crops

Essential for maximizing soil productivity is controlling pathogens and increasing the abundance of microorganisms that confer beneficial traits. Various microbial strains belonging to the genera *Bacillus, Pseudomonas*, *Trichoderma*, *Paenibacillus*, *Burkholderia*, *Isaria*, and *Metarhizium* were found to be effective biocontrol agents against *Fusarium* spp. (Dong et al., 2023), *Erwinia amylovora* (Sharifazizi et al., 2017), *Botrytis cinerea* (Toral et al., 2020), *Verticillium dahliae* (Dhouib et al., 2019), *Phytophthora* spp. (Syed-Ab-Rahman et al., 2018), *Sclerotium rolfsii* (Chen et al., 2020), *Rosellinia necatrix* (Tienda et al., 2020), *Sclerotinia sclerotiorum* (Duke et al., 2017), *Alternaria alternata* (Ji et al., 2021), *Pythium ultimum*, *Rhizoctonia solani* (Chávez-Ramírez et al., 2020), *Colletotrichum* spp. (Han et al., 2015; Kim et al., 2021), and insect pests (Brunner-Mendoza et al., 2019). Application of biofertilizers stimulated the abundance of indigenous, potentially beneficial microbial taxa, including members of *Bacillus*, *Burkholderia*, *Rhizobium*, *Streptomyces*, *Pseudomonas*, *Leptosphaeria*, *Frankiales*, *Xanthomonadales*, *Sphingobacteriales*, *Trichoderma* and *Mortierella* (Dong et al., 2023; Shen et al., 2015; Mao and Jiang, 2021; Qi et al., 2022), all of which are essential components of soil microbiomes. The potential of biocontrol agents (BCAs) including beneficial bacteria and to suppress pathogens and promote plant health has been widely demonstrated, yet findings across studies remain inconsistent and system-dependent. While numerous reports confirm their effectiveness against soil-borne and foliar diseases through mechanisms such as antibiosis, induced systemic resistance (ISR), and nutrient competition (O’Brien, 2017; Köhl et al., 2019), other studies reveal variable efficacy under field conditions, often due to differences in soil microbiome composition, environmental factors, and pathogen pressure (Compant et al., 2005). For instance, *Trichoderma* and *Bacillus* strains that perform well in controlled experiments may fail to establish or persist in soils with high microbial competition or under extreme temperatures (Woo et al., 2014; Mitter et al., 2017). The context specificity of BCAs is further influenced by plant genotype, cultivation practices, and climate, all of which modulate root exudate chemistry and microbial colonization (Backer et al., 2018; Fira et al., 2018). Moreover, while some BCAs enhance plant growth and disease suppression, others may exhibit neutral or even antagonistic interactions with native microbiota, reducing overall ecosystem stability (Berg et al., 2017). These contradictions underscore the need for systems-based biocontrol strategies that integrate multi-omics data, microbial consortia design, and predictive ecological modeling to ensure consistent and sustainable crop protection outcomes across diverse agroecosystems.

#### 3.1.3 Soil enzymatic activity as a key player in maintaining quality properties and fertility

The intensity of soil processes depends strongly on the activity of microorganisms, which synthesize a wide range of enzymes involved in the breakdown of complex carbon, nitrogen, and phosphorus compounds (Luo et al., 2020). It has been documented that soil inoculated with biofertilizers exhibited increased activity of β-glucosidase, protease, dehydrogenase, phosphomonoesterases, urease, invertase, nitrogenase, and catalase (Dal Cortivo et al., 2020; Mącik et al., 2020b; Nosheen et al., 2018; Madhaiyan et al., 2010; Krey et al., 2011; Srivastava and Singh, 2021; Mao and Jiang, 2021). Elevated soil enzymatic activity is associated not only with increased rates of matter turnover and energy flow but also with higher nutrient bioavailability, which is indispensable for plant growth (Jacoby et al., 2017). Concerning soil nutrient levels, utilization of nitrogen-fixing, phosphate, and potassium solubilizing/mobilizing microorganisms increased nutrient uptake in agriculturally essential plants, including wheat (Wang et al., 2020), soybean (Egamberdieva et al., 2017), maize (Ribeiro et al., 2022), sugarcane (Rosa et al., 2020), and tomato (Pokluda et al., 2021). Moreover, the total relative abundance of genes involved in nitrogen metabolism, inorganic P-solubilization, and organic P-mineralization increased in soil treated with biofertilizers (Wang G. et al., 2021). PICRUSt analyses of bacterial community functional profiles showed that soil amended with biofertilizers had more significant numbers of operational taxonomic units (OTUs) associated with amino acids and lipid transport and metabolism, energy production and conversion (Qi et al., 2022; Mącik et al., 2020b), coenzyme transport and metabolism, signal transduction mechanisms (Tian et al., 2022), glycan biosynthesis and metabolism (Liu et al., 2021), xenobiotics biodegradation and metabolism (Wang G. et al., 2021), and P cycling (Mącik et al., 2022).

Biofertilizers may also play a role in adaptation to climate change. A meta-analysis by Rubin et al. (2017) found that plant growth-promoting rhizobacteria (PGPR) can mitigate drought stress, improving root and shoot mass and reproductive yield. Global warming promotes the occurrence of new phytopathogens (Velásquez et al., 2018), and, as described above, biofertilizers may constitute a powerful tool against plant diseases of microbial origin. Furthermore, overloading soil with chemical mineral fertilizers exacerbates GHG emissions (Chai et al., 2019). One solution to alleviate these emissions and, concomitantly, to reduce mineral fertilizer quantities may be biological nitrogen fixation (BNF) provided by bacteria, including *Rhizobium* spp., *Azotobacter* spp., and *Azospirillum* spp. It has been estimated that up to 70% of global crop N uptake can be attributed to BNF (Kuan et al., 2016). It is also worth emphasizing that *Rhodopseudomonas palustris* reduced CH_4_ emissions in rice paddies by 24–28% (Kantachote et al., 2016). Carbon sequestration decreases atmospheric CO_2_ concentration and increases soil organic carbon (SOC) content (Hu et al., 2018). One approach to support soil carbon sequestration is biofertilization. The application of biofertilizers was found to increase SOC stocks in studies conducted by Dębska et al. (2016), Adak et al. (2014), Shukla et al. (2017), Yadav et al. (2016), Ansari and Mahmood (2017), Thilagar et al. (2016), and Yilmaz and Sönmez (2017). Moreover, as mentioned above, mineral fertilizer enriched with beneficial fungal strains increased the abundance of the cbbLR and pmoA genes, which are essential for carbon storage in soil (Pertile et al., 2021). In general, SOC content can be improved through the following mechanisms: accelerating organic matter humification, increasing the photosynthetic activity of biofertilizers containing microalgae and cyanobacteria, and promoting plant growth and the incorporation of organic matter through plant roots (Food and Agriculture Organization of the United Nations [FAO] and ITPS, 2021). Soil enzymatic activity plays a central role in maintaining soil quality, fertility, and ecosystem function, yet findings across studies are inconsistent under different management and environmental conditions. Enzymes such as phosphatases, dehydrogenases, and ureases are crucial indicators of nutrient cycling and microbial activity, but their sensitivity to soil type, organic matter content, moisture, and pH often leads to variable interpretations of soil health (Nannipieri et al., 2018; Burns et al., 2013). For instance, agricultural intensification and fertilizer regimes can either stimulate or inhibit enzymatic activity depending on substrate availability and microbial community structure (Allison et al., 2008; Trivedi et al., 2016b). Similarly, climate factors such as temperature fluctuations and drought stress can alter enzyme kinetics and stability, influencing nutrient turnover and plant productivity (German et al., 2012). These inconsistencies highlight the need for context-specific assessment frameworks that integrate enzymatic activity with microbiome composition, soil physicochemical data, and land-use history to accurately evaluate soil fertility and sustainability.

### 3.2 Managing plant microbiome

#### 3.2.1 Beneficial microorganisms’ mode of action

Beneficial microorganisms can influence plants in numerous ways. Applying carefully selected microbial inoculants to a plant-growing site can enhance plants’ resilience to biotic and abiotic stresses and improve soil quality. This increases the availability of micro- and macro-elements to plants and the soil’s organic matter content (Pylak et al., 2019). Their application can also impact soil and plant health by shifting and maintaining the stability of soil microbiomes (Frąc et al., 2018).

Bacteria and fungi that possess properties that inhibit the growth of plant pathogens, stimulate plant growth, or positively affect soil quality are called “beneficial,” and, after conducting sufficient research, they might be applied to crops. Locally occurring beneficial microorganisms can most effectively inhibit the growth of locally occurring pathogens as they are already adapted to local conditions (Fikri et al., 2018). Many bacterial strains belonging to the genera of *Pseudomonas*, *Bacillus*, and *Actinobacteria* can inhibit the growth of fungal plant pathogens such as *Fusarium* sp., *Botrytis* sp., *Phytophthora* sp., *Verticillium* sp., and *Colletotrichum* sp., which are some of the most essential fungal and fungal-like plant pathogens that can lead to 40% yield loss in crops (Saeed et al., 2021). Applying appropriate microorganisms as biopreparations can help plants cope with biotic stresses.

The mode of action of microbial inoculants is complex. It can be divided into five main categories: inactivation of pathogen enzymes, competition for nutrients and space, mycoparasitism, production of inhibitory compounds, and induced resistance (Elad et al., 1999; Haran et al., 1996; Lorito et al., 1996; Roco and Pérez, 2001; Yedidia et al., 1999; Yedidia et al., 2000; Oszust et al., 2020). Highly competitive beneficial microorganisms quickly colonize environmental niches, preventing other potentially harmful organisms from establishing themselves (Poonam, 2021). Both beneficial bacteria and fungi can secrete antibiotic compounds such as glovirin, colicin, nisin, and mycobacillin, produced by *Trichoderma* sp., *Lactococcus lactis*, *Burkholderia* sp., and *Pseudomonas* sp., respectively, which might inhibit the growth of pathogenic fungi and bacteria (Elshahat et al., 2016; Mohanty et al., 2021; Zimina et al., 2020). Mycoparasitism is a mode of action characteristic of beneficial fungi that grow toward other fungal hyphae and coil around them, simultaneously producing lytic enzymes that degrade the other (pathogenic) fungi’s cell walls (Rajani et al., 2021). Furthermore, some strains of beneficial fungi, such as *Trichoderma harzianum* T39, can produce proteases, i.e., endopolygalacturonase (endo-PG) or pectate lyase, which inactivate pathogenic enzymes that can hydrolyze plant tissues (Roco and Pérez, 2001; Elad, 2000). Some plant growth-promoting rhizobacteria and arbuscular mycorrhizal fungi can induce systemic resistance to plant pathogens (Harel et al., 2011) and improve plant growth and vitality (Ważny et al., 2021; Ważny et al., 2022). *Trichoderma harzianum* T39 applied to the soil induced systemic resistance in strawberry plants attacked by powdery mildew caused by *Podosphaera aphanis* and inhibited the pathogen’s growth (Harel et al., 2011). In this case, plants were triggered to produce antifungal compounds or lytic enzymes, such as chitinase, to inhibit pathogen infection.

#### 3.2.2 Environmental stress resistance improvement

Unlike biotic stress, abiotic stress is caused by environmental factors such as extreme temperatures, salinity, drought, acidic soil, and metal toxicity. Globally increasing temperatures and decreasing water availability will increase drought risk. Microbial inoculants, especially PGPR, can produce bacterial phytohormones such as auxins, gibberellins, cytokinins, ethylene, and abscisic acid (ABA). These hormones can increase the number of root tips and root surface area, influence water loss regulation by controlling stomatal closure, and affect stress signal transduction pathways. PGPRs can also produce exopolysaccharides (EPS) that coat plant roots and protect them from desiccation during drought periods. Proper plant hydration and water management can also help plants handle high temperatures (Kaushal and Wani, 2016). Excessive soil salinity affects approximately 20% of the world’s total cultivated area. Since water used for irrigation often contains higher-than-preferred salt levels, up to 50% of irrigated land globally is exposed to excessive soil salinity. This also negatively affects soil water potential and availability, leading to cellular dehydration. Acidic soils are often associated with changes in the solubility of metal ions and nutrient availability. Aluminum toxicity in the soil is considered a limiting factor for plant growth, as it inhibits root growth. Furthermore, aluminum forms complexes with phosphoric acid in acidic soils, making it unavailable for plant assimilation. However, many PGPR can enhance phosphorus solubilization and bind aluminum ions by forming Al^3+^-siderophore complexes. Presented mechanisms have a favorable effect on plants’ resilience to environmental stressors and pathogens. Healthy plants free of pathogens can cope with environmental stresses and produce more reliable and higher yields (Dutta and Bora, 2019).

Reversing the current trend of soil biodiversity degradation is one of the most critical agricultural targets for the future (EU Communication, 2019). Microbial supplementation can improve soil quality, health, and biodiversity. Bacteria belonging to the *Rhizobium* and *Pseudomonas* genera can fix atmospheric nitrogen and increase its concentration in soil by converting it to other nitrogen compounds. As previously described, microorganisms can increase the concentrations of organic and inorganic phosphorus and sulfur available to plants, thereby enhancing soil organic matter (Prasad et al., 2015; Figueiredo et al., 2013; Shah et al., 2021). Increased soil organic matter content leads to better water retention, improves soil fertility by providing cation-exchange sites, and provides a reserve of plant macro- and micro-nutrients slowly released from soil organic matter (Yavitt et al., 2021).

Some currently commercially available biopreparations for farmers consist of different microorganisms, such as *Pythium oligandrum* spores, arbuscular mycorrhizal fungi (including *Glomus* sp.), *Trichoderma viride*, and rhizosphere bacterial species (*Bacillus subtilis*, *Pseudomonas fluorescens*, and *Streptomyces* spp.). These strains can reduce environmental stresses and enhance the growth of apple and sour cherry trees (Grzyb et al., 2015). Moreover, biopreparations containing *Trichoderma* species can reduce infection rates of *Fusarium* sp., *Rhizoctonia* sp., and *Pythium* sp. on various plants (Woo et al., 2014; Oszust et al., 2020; Oszust et al., 2021). Plant growth-promoting bacteria and arbuscular mycorrhizal fungi can produce exudates that bind toxic metal ions and increase the availability of micro- and macronutrients.

Beneficial microorganisms, including bacteria and fungi, may affect and enhance plant resilience to biotic and abiotic stresses. Means for these effects include improving soil quality and increasing nutrient availability. Said microorganisms possess various mechanisms of action, such as competition for nutrients and space, production of inhibitory compounds, mycoparasitism, inactivation of pathogen enzymes, and induced systemic resistance, thereby contributing to more reliable and higher yields. Moreover, beneficial microorganisms may find application in novel farming methods, such as growing superfoods, vertical farming, and urban farming.

Improving environmental stress resistance through microbiome-based and ecological strategies has become a key focus in sustainable agriculture, yet outcomes remain highly variable across crops, soils, and climates. Beneficial microorganisms such as *Pseudomonas*, *Bacillus*, and arbuscular mycorrhizal fungi enhance plant tolerance to drought, salinity, and temperature extremes by modulating antioxidant activity, osmolyte accumulation, and hormonal balance (Yang et al., 2009; Niu et al., 2018). However, the effectiveness of these microbial inoculants often depends on soil nutrient status, native microbial diversity, and host genotype, leading to inconsistent field performance (Sessitsch et al., 2019; Compant et al., 2019). Environmental fluctuations further influence microbial persistence and signaling, sometimes reducing the long-term benefits observed under controlled conditions (Timmusk et al., 2017; Trivedi et al., 2020). Consequently, integrating stress-resilient microbiomes with adaptive management practices and predictive ecological modeling is crucial for developing reliable strategies that sustain crop productivity under changing climatic scenarios.

### 3.3 How microbes interact with each other – plant–soil–microbiome networks

As Berg et al. (2020) described, microbes form networks and interact with one another, resulting in diverse consequences for microbial fitness, dynamics, and functional capacities within the microbiome. Coban et al. (2022) proposed that microbial communities serve as indicators of ecosystem health and restoration, as well as agents in the recovery of degraded soil. The microbiome improves plant fitness and can increase plant resilience and nutrient mobilization (Berg et al., 2016; Hirsch and Mauchline, 2012; Yadav et al., 2021). The composition of the plant microbiome depends on plant species and root exudates (Sasse et al., 2017), soil type (Mącik et al., 2020b), and also the cultivar of a given plant species (Panek et al., 2021; Vink et al., 2021b). The soil microbiome is the primary source from which plants recruit beneficial microbes as partners in interactions; both its structure and diversity are increasingly recognized as significant contributors to plant health enhancement and sustainable agricultural development (Vink et al., 2021a). Therefore, microbiome-based strategies for plant breeding crucially include traits that interact with the microbiome when selecting resistant varieties for specific climate conditions and for resilience to biotic and abiotic stresses (Dubey et al., 2019; Coban et al., 2022). Moreover, microorganism inoculants can reshape soil microbiomes (Liu et al., 2022). They can interact with native soil microbial communities (Mawarda et al., 2020) and communicate indirectly through plant root exudates (Cesari et al., 2019). Microbial inoculation can cause tremendous shifts in the number and composition of soil microbiomes, but its impact depends on the techniques applied (Trabelsi and Mhamdi, 2013).

In summary, soil and plant microbiomes play a pivotal role in maintaining the health of plant and soil ecosystems and enhancing crop productivity. Interactions between the soil and plant microbiomes maintain and shape soil properties and quality. Recently, the effects of cooperation among plant-microbial communities on plant growth and health have become increasingly important. The services provided by plant-associated microorganisms can shape the plant host’s natural immunity (Vannier et al., 2019). This interaction is so beneficial to plants that they actively attract soil microorganisms by secreting compounds that stimulate their growth (Reinhold-Hurek et al., 2015; Sasse et al., 2017). However, a more holistic approach is needed to better understand the relationships between microbes and plants. Currently, the challenges are to define microbial-plant–soil interactions as whole communities and to design microbial consortia to balance rhizosphere communities, thereby simultaneously protecting plants, enhancing nutrient availability, and ensuring the stability of soil microbiomes, thereby mitigating the impact of biotic and abiotic stressors caused by a changing climate.

## 4 Innovative agricultural approaches

The challenges posed by broad urbanization, a changing climate, and a rapidly growing global population, which are driving increasing demand for food, have led to the development of new approaches to innovative farming, such as vertical and urban agriculture. Vertical farming involves cultivating crops in containers stacked vertically within controlled environments, employing technologies such as media-based methods, hydroponics, aeroponics, and LED lighting. Such an approach allows optimization not only of space but also of water and nutrient use through the application of closed-loop systems, minimizing reliance on herbicides and pesticides and further improving sustainability and resource efficiency (Benke and Tomkins, 2017). On the other hand, urban farming integrates farming into metropolitan areas, such as transforming rooftops, vacant spaces, or indoor areas into farmland. Such urban farming can enhance food security and resilience against disruptions to the food supply chain, while promoting community engagement and environmental conservation, or improving urban landscapes (Specht et al., 2014; Thomaier et al., 2015).

Another topic closely related to urban and vertical farming is the production of so-called “superfoods.” Often, foods such as sprouts and microgreens are considered superfoods. This food is rich in macro- and micronutrients and possesses favorable properties and effects on human health, thanks to its high content of vitamins, minerals, and antioxidants (Franco Lucas et al., 2021). The integration of a controlled vertical farming environment with superfood production ensures consistent quality and yield, maintaining year-round availability regardless of external conditions. Moreover, urban agriculture can significantly reduce the carbon footprint associated with food storage and transportation by lowering logistics requirements (Kulak et al., 2013). As such, monitoring and maintaining the microbiome composition of vertically and urban-cultivated plants is even more critical, as the stability of the plant holobiont ensures the predictability and stability of production and resilience against plant diseases (Du et al., 2025).

## 5 Sequencing methods and bioinformatic tools as relevant approaches to determine soil microbiome changes for the prediction of plant diseases, soil health, and quality in a changing climate

Climate change impacts significant aspects of our lives. But not only is human life altered. Rising temperatures and CO_2_ levels alter the soil microbiome’s abundance, behavior, and diversity, affecting its fertility and soil–plant microbiome interactions, which, in turn, change plant resistance to stresses and vulnerability to crop diseases. Currently, the most efficient methods for studying changes in soil and plant microbiome diversity involve sequencing and characterizing microbial DNA. Advances in DNA sequencing methods, computer power, and bioinformatic tools have enabled the study of the genetic diversity of microbial communities, including previously uncultivable species. Two strategies for determining microbiome composition have emerged: amplicon and metagenomic sequencing. Amplicon sequencing focuses on defining and amplifying the sequence of a single gene fragment. Most frequently targeted are marker genes that are taxonomically and phylogenetically informative (Hugenholtz and Pace, 1996; Schoch et al., 2012). Such sequencing is often referred to as metataxonomics (Marchesi and Ravel, 2015). The relatively low cost of metataxonomic methods allows for studying how microbial profiles and their diversity change in response to environmental changes. Although this approach is promising and accessible, it has some critical constraints. First, amplicon sequencing is prone to PCR-related bias, including artifacts that skew the distribution of PCR products due to unequal amplification (Acinas et al., 2005; Hong et al., 2009). Moreover, choosing primers is crucial for obtaining high-quality data and is constantly being re-evaluated.

In contrast to amplicon sequencing, metagenomic sequencing sequences all the DNA present in a studied sample. This is done using a method known as “shotgun” metagenomics. Genetic material from a community is cut into shorter fragments using various techniques (such as sonication, mechanical tearing, or enzymatic restriction) and, after adding sequencing adapters, sequenced (Sharpton, 2014). Metagenomic sequencing can provide more comprehensive information about the genes involved in metabolic pathways in a sample, as well as generate metagenome-assembled genomes (MAGs). However, shotgun sequencing, especially of soil samples, requires a sequencing depth that is not comparable to that of amplicon sequencing. Sequencing of microbiomes is heavily hindered by host DNA “contamination,” requiring even greater sequencing depth (Sharpton, 2014).

To study shifts in soil microbiomes driven by climate change, appropriate sequencing techniques must be used. Illumina sequencing-by-synthesis is currently the gold standard in metataxonomics due to its ability to sequence either the V4 region of the 16S rRNA gene or the V3–V4 region of the 16S rRNA gene, as well as ITS1 or ITS2, in large quantities. Due to the observation that the longer the read across marker region, the better the accuracy of microbial identification, developments in recent years have brought different approaches to metataxonomics, focusing on obtaining much longer reads (Payne et al., 2019; Wang Y. et al., 2021), spanning the entire 16S rRNA gene (Wagner et al., 2016) or the entire ITS1-5.8S-ITS2 region (Purahong et al., 2019). Although there have been early-stage problems with read quality (Goodwin et al., 2016; Tedersoo et al., 2018), there already is the possibility to utilize customer-ready third-generation long-read sequencing technologies, with PacBio offering Single-Molecule-Real-Time (SMRT) sequencing within its latest Revio and Vega platforms, while Oxford Nanopore Sequencing Technologies offers nanopore sequencing-based technology with both mobile and accessible platform Minion and higher throughput ones like Gridion and Promethion sequencers (Goodwin et al., 2016; Tedersoo et al., 2018).

Thanks to the integration of metataxonomic and metagenomics approaches enabled by The Data Integration Analysis for Biomarker discovery using a Latent cOmponents (DIABLO) framework (Singh et al., 2019) in mixOmics (Rohart et al., 2017), with zero radius Operational taxonomic units (zOTU) constructed by USEARCH (Edgar, 2010) and Ribosomal Database Project (Cole et al., 2014), while also using Metagenomic Rapid Annotations using Subsystems Technology (MG-RAST), Ferrarezi et al. (2023) confirmed positive effect of plant growth promoting bacteria *Azospirillum brasilense* inoculations on maize growth. Moreover, a metagenomics metastudy by Masuda et al. (2024) found that Anaeromyxobacteraceae and Geobacteraceae within Deltaproteobacteria are groups of nitrogen-fixing microorganisms present within microbiomes in ecosystems with different land usage types and geographic origins. Functional profiling enabled by the metagenomics approach to soil studies allowed Pang et al. (2021) to determine that continuous sugarcane cultivation significantly reduction the abundance of the functional pathway, such as genes related to nitrogen and sulfur cycling in soil, reduced diversity of soil bacterial and fungal communities, significantly reduced the number of bacteria associated with soil nitrogen and sulfur cycling functions, and enriched pathogenic bacteria.

Recent years have brought significant advances in computational power, making it possible to apply supervised Machine Learning (ML) techniques to studies of soil. ML is widely used with microbiome data, as naive Bayesian classifiers have found utility in assigning taxonomy to Amplicon Sequence Variants or Operational taxonomic Units (Pedregosa et al., 2011; Wang et al., 2007), while model for learning error profile of reads within sequencer run based on LOESS (locally estimated scatterplot smoothing) function fitting was developed to overcome limitations of OTU clustering with emergence of ASV approach to metataxonomic data. Moreover, methods such as the random forest (RF) classifier and the L2 Support Vector Machine (L2-SVM) with a linear kernel have been successfully deployed to predict many soil properties and even crop productivity (Chang et al., 2017; Wilhelm et al., 2022).

What is essential is that the RF method was found to perform best for typical microbiome data (Thompson et al., 2019; Zhou and Gallins, 2019), while L2-SVM is often chosen for its speed (Topçuoğlu et al., 2020). Depending on the choice of ML algorithm, classifier, or regressor, categorical and numerical variables can be predicted.

Recent advances in metagenomics, metatranscriptomics, and metabolomics have revolutionized our understanding of plant-microbe interactions, enabling the identification of functional genes, signaling pathways, and metabolic exchanges that govern plant health and soil ecosystem dynamics (Levy et al., 2018; Hacquard et al., 2015). These multi-omics approaches reveal not only the taxonomic composition but also the functional potential and real-time activity of microbial communities in response to environmental and plant-derived cues (Delgado-Baquerizo et al., 2018). Integrating these data with artificial intelligence (AI) and machine learning (ML) has enabled researchers to build predictive models that link microbiome composition to plant traits, stress tolerance, and disease outcomes (Xu et al., 2018). For instance, ML algorithms are now applied in disease prediction, microbiome-based crop yield forecasting, and trait selection for breeding programs that favor beneficial microbial associations (Xu et al., 2018; Zhao et al., 2023; Chang et al., 2017). Together, these approaches are transforming plant–microbiome research from descriptive ecology into predictive and actionable systems biology, paving the way for precision agriculture and microbiome engineering.

**Figure 2:**
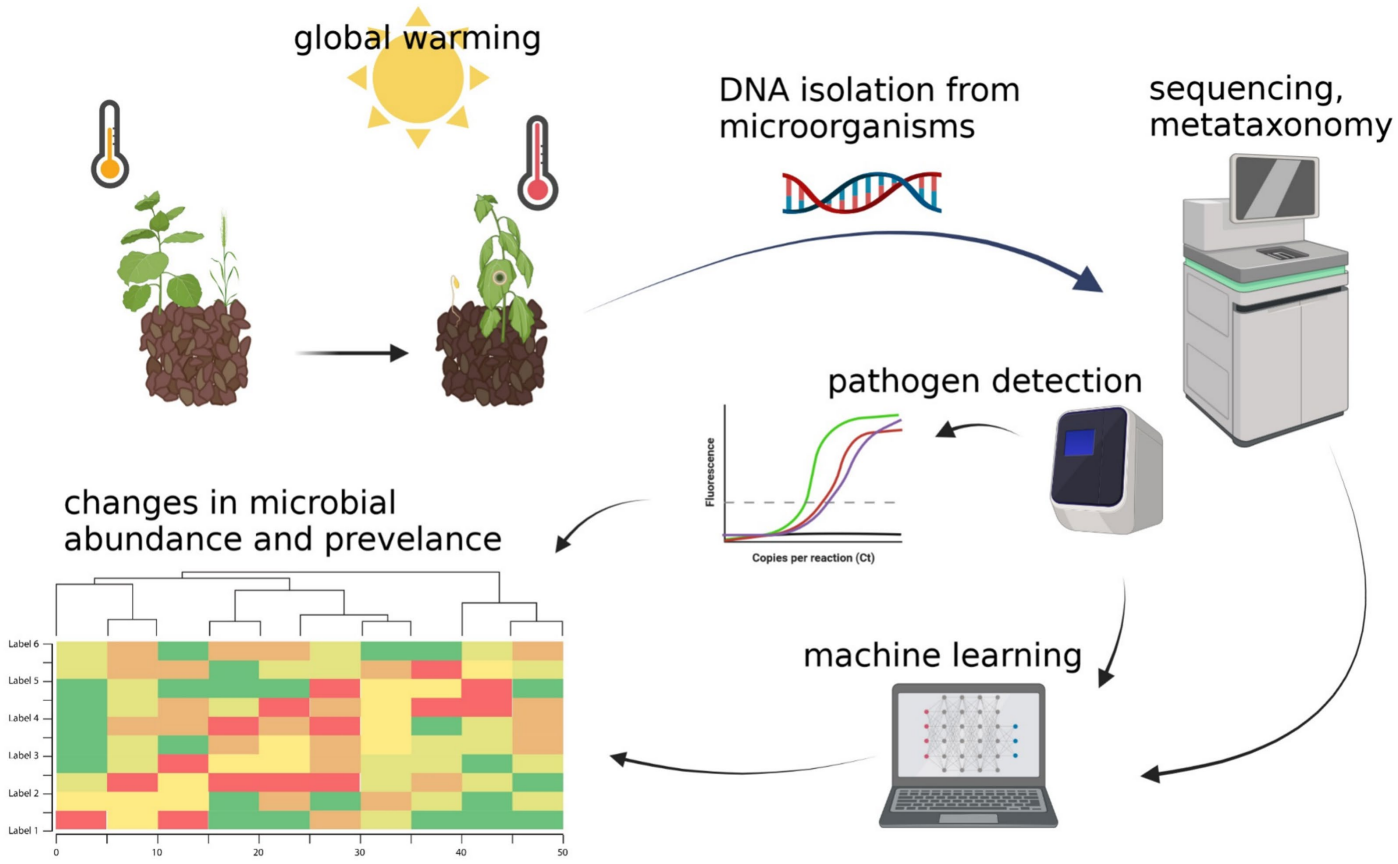
Assessment of microbial community structure and prediction of plant diseases, soil health, and quality in a changing climate based on soil microbiome analysis and computation. Created with BioRender.com.

Machine learning algorithms can now be easily employed using QIIME2 plugin “*sample-classifier*,” which supports supervised ML methods for classification and regression of sample properties (Bolyen et al., 2019; Bokulich et al., 2018). There are also attempts to utilize soil and plant metagenomic data, along with machine learning, to predict their susceptibility to plant diseases. It is a powerful tool that may become useful in predicting how microbiomes will adapt to climate change and how these changes will affect soil properties and health (Figure 2). Recently, Demilie (2024) suggested that the best results for predicting plant diseases are obtained when deploying ML models on each compartment of the studied plant and its environment. It is also stated that new approaches, such as the real-time application of ML methods to predict or detect diseases, are being developed, and that studies focusing on these approaches should be encouraged. Bioinformatics can be deployed to assess microbial community structure and functions, predict plant diseases, and assess soil health and quality in a changing climate, based on soil microbiome analysis and computation.

### 5.1 Current limitations and prospects for future studies

Metagenomics is a crucial approach for gathering information on microbial communities in soil. However, whether this approach provides an accurate representation of soil microbial diversity remains a question. Soil represents a vast genetic reservoir; unfortunately, despite new concepts and technological advancements, the diversity and functionality of soil microbes remain largely unknown, mainly because of the complex soil structure, which poses difficulties for extracting DNA thoroughly and efficiently. Additionally, cell adsorption and DNA adherence to soil components result in losses of genetic information. Therefore, current DNA exploitation techniques allow the study of mainly dominant microbial populations in soil (Lombard et al., 2011). However, researchers have recently become increasingly interested in the rare microbiota present in soil, which appears to be a promising direction for future study.

Metagenomic research generates enormous amounts of data, posing significant challenges for sequence assembly and analysis. Technological advances have made it easier to collect vast amounts of sequence data. However, soil samples with thousands of unique taxa are frequently poorly described and characterized (Anthony et al., 2024). Although improved strategies for investigating DNA/RNA in soil enable an understanding of microbial diversity, interpreting the limited data is challenging, and a detailed catalog of soil microorganisms and functional genes does not exist for any soil (Kaushik et al., 2020).

Therefore, there is a need to move from the microbes list present in the samples to their functionality and involvement in soil processes (Frąc et al., 2018; Frąc et al., 2022). Moreover, the challenge is to identify appropriate DNA/RNA extraction techniques that allow distinguishing active, dormant, dead, and total microorganisms within the soil microbial community (Blagodatskaya and Kuzyakov, 2013).

Finally, as microbial communities are essential components of soil ecosystems and metagenomics data are increasingly easy to collect, key challenges of soil metagenomics studies include soil physicochemical properties causing difficulties in genetic material extraction, selecting natural controls as a good baseline, as well as sharing data, allowing meta-analyses crucial for overall effects investigation (Leite et al., 2022).

## 6 Conclusion and future directions

Increasing soil performance, persistence, and inoculation efficiency with microbial-based products is a priority to harness their potential and reduce risks of adverse outcomes. Although numerous studies have focused on aspects of soil microbiomes, including structure, biodiversity, and functions, significant knowledge gaps remain, and substantial research is needed to understand the interactions within soil–plant–microbiomes in different ecosystems, thereby gaining a deeper understanding of soil functionality and microbiome-based services for agroecosystems. The challenges in this context can serve as principal future perspectives in soil microbiome research, especially under changing climate conditions. In conclusion, we suggest studies to include (a) a deeper explanation of how soil microbiomes’ structure and functionality can contribute to the future development of climate-resilient and resistant plants to biotic and abiotic stresses; (b) integration of different omics approaches to increase higher-resolution characterization of soil microbiomes to define holistic healthy (eubiotic) and unhealthy (dysbiotic, pathobiomic) plant and soil microbiomes both to develop predictive models of plant disease occurrence and to improve climate models; (c) implementation of more cultivation-based assays into microbiome research to more explicitly describe ecotypes and adaptation modes of specific microbial groups to a changing environment; to date, there are not well enough described and designed studies in these areas. These prospects are supported by the latest soil priorities in the European Union, which highlight soil health restoration as an essential challenge by 2050. These priorities are contained in the Horizon Europe mission “Caring for soil is caring for life”, supported by “A Soil Deal for Europe”, and contribute to multiple European Green Deal targets on climate resilience, sustainable farming, zero pollution, and biodiversity. Moreover, the strategies and approaches presented in this review align with *the European Commission’s Microbiome World Pathway*, which identifies systemic challenges and necessary actions by 2030 (European Commission, 2020).

Finally, given the challenges facing modern and future agriculture, revolutionary approaches and solutions to climate and sustainability problems, food production, security, and plant health defense must be pursued. The methods, strategies, and challenges should not only refer to large-scale agricultural and horticultural crops, but also range from agroecological management techniques based on microbially mediated ecosystem functions to advanced sustainable farming techniques, such as vertical and urban farming, and the production of superfoods, including microgreens. The application of microbial inoculants, biofertilizers, and genetically enhanced biocontrol agents offers significant promise for sustainable agriculture, yet it also raises ecological and regulatory concerns that warrant careful consideration. Introducing non-native or engineered microorganisms into soil ecosystems can disrupt native microbial community structure and function, potentially leading to competitive exclusion or altered nutrient cycling (Li et al., 2022; Müller et al., 2016). Moreover, the risk of horizontal gene transfer (HGT) between introduced strains and indigenous microbes poses challenges for biosafety, as antibiotic resistance or virulence genes could be unintentionally disseminated (van Elsas et al., 2003; Heuer and Smalla, 2012). Environmental persistence of inoculants and their metabolites may also cause unforeseen ecological feedbacks, particularly under varying soil and climate conditions (Mumtaz et al., 2025). Regulatory frameworks for microbial-based products remain fragmented globally, often lagging behind technological advances in synthetic biology and microbial engineering (Elazzazy et al., 2025; Pellegrini et al., 2025). Therefore, risk assessment protocols integrating genomic, ecological, and functional data are essential to ensure that microbiome-based innovations promote sustainability without compromising ecosystem integrity or biosafety.

## References

[ref1] Acet Ö. Erdönmez D. Acet B. Ö. Odabaşı M. (2021). N-acyl homoserine lactone molecules assisted quorum sensing: effects consequences and monitoring of bacteria talking in real life. Arch. Microbiol. 203, 3739–3749. doi: 10.1007/s00203-021-02381-9, 34002253

[ref2] Acinas S. G. Sarma-Rupavtarm R. Klepac-Ceraj V. Polz M. F. (2005). PCR-induced sequence artifacts and Bias: insights from comparison of two 16S rRNA clone libraries constructed from the same sample. Appl. Environ. Microbiol. 71, 8966–8969. doi: 10.1128/AEM.71.12.8966-8969.2005, 16332901 PMC1317340

[ref3] Adak T. Singha A. Kumar K. Shukla S. Singh A. Singh V. K. (2014). Soil organic carbon, dehydrogenase activity, nutrient availability and leaf nutrient content as affected by organic and inorganic source of nutrient in mango orchard soil. J. Soil Sci. Plant Nutr. 14, 394–406. doi: 10.4067/S0718-95162014005000031

[ref4] Agler M. T. Ruhe J. Kroll S. Morhenn C. Kim S. T. Weigel D. . (2016). Microbial hub taxa link host and abiotic factors to plant microbiome variation. PLoS Biol. 14:e1002352. doi: 10.1371/journal.pbio.1002352, 26788878 PMC4720289

[ref5] Allison S. D. Czimczik C. I. Treseder K. K. (2008). Microbial activity and soil respiration under nitrogen addition in Alaskan boreal forest. Glob. Change Biol. 14, 1156–1168. doi: 10.1111/j.1365-2486.2008.01549.x

[ref6] Alori E. T. Babalola O. O. (2018). Microbial inoculants for improving crop quality and human health in Africa. Front. Microbiol. 9:2213. doi: 10.3389/fmicb.2018.02213, 30283427 PMC6156547

[ref7] Ansari R. A. Mahmood I. (2017). Optimization of organic and bio-organic fertilizers on soil properties and growth of pigeon pea. Sci. Hortic. 226, 1–9. doi: 10.1016/j.scienta.2017.07.033

[ref8] Anthony W. E. Allison S. D. Broderick C. M. Rodriguez L. C. Clum A. Cross H. . (2024). From soil to sequence: filling the critical gap in genome-resolved metagenomics is essential to the future of soil microbial ecology. Environ. Microbiome 19:56. doi: 10.1186/s40793-024-00599-w, 39095861 PMC11295382

[ref9] Backer R. Rokem J. S. Ilangumaran G. Lamont J. Praslickova D. Ricci E. . (2018). Plant growth-promoting rhizobacteria: context, mechanisms of action, and roadmap to commercialization of biostimulants for sustainable agriculture. Front. Plant Sci. 9:1473. doi: 10.3389/fpls.2018.01473, 30405652 PMC6206271

[ref10] Bahram M. Hildebrand F. Forslund S. K. Anderson J. L. Soudzilovskaia N. A. Bodegom P. M. . (2018). Structure and function of the global topsoil microbiome. Nature 560, 233–237. doi: 10.1038/s41586-018-0386-6, 30069051

[ref11] Banerjee S. Schlaeppi K. van der Heijden M. G. (2018). Keystone taxa as drivers of microbiome structure and functioning. Nat. Rev. Microbiol. 16, 567–576. doi: 10.1038/s41579-018-0024-1, 29789680

[ref12] Barberán A. Bates S. T. Casamayor E. O. Fierer N. (2012). Using network analysis to explore co-occurrence patterns in soil microbial communities. ISME J. 6, 343–351. doi: 10.1038/ismej.2011.119, 21900968 PMC3260507

[ref13] Bashan Y. de-Bashan L. E. Prabhu S. R. Hernandez J. P. (2014). Advances in plant growth-promoting bacterial inoculant technology: formulations and practical perspectives (1998–2013). Plant Soil 378, 1–33. doi: 10.1007/s11104-013-1956-x

[ref14] Bender S. F. Wagg C. van der Heijden M. G. A. (2016). An underground revolution: biodiversity and soil ecological engineering for agricultural sustainability. Trends Ecol. Evol. 31, 440–452. doi: 10.1016/j.tree.2016.02.016, 26993667

[ref15] Benke K. Tomkins B. (2017). Future food-production systems: vertical farming and controlled-environment agriculture. Sustain Sci Pract Policy 13, 13–26. doi: 10.1080/15487733.2017.1394054

[ref16] Berg G. Köberl M. Rybakova D. Müller H. Grosch R. Smalla K. (2017). Plant microbial diversity is suggested as the key to future biocontrol and health trends. FEMS Microbiol. Ecol. 93:fix050. doi: 10.1093/femsec/fix050, 28430944

[ref17] Berg G. Rybakova D. Fischer D. Cernava1 T. Vergès M. C. C. Charles T. . (2020). Microbiome definition re-visited: old concepts and new challenges. Microbiome 8:103. doi: 10.1186/s40168-020-00875-0, 32605663 PMC7329523

[ref18] Berg G. Rybakova D. Grube M. Köberl M. (2016). The plant microbiome explored: implications for experimental botany. J. Exp. Bot. 67, 995–1002. doi: 10.1093/jxb/erv466, 26547794 PMC5395086

[ref19] Bhattacharyya P. N. Jha D. K. (2012). Plant growth-promoting rhizobacteria (PGPR): emergence in agriculture. World J. Microbiol. Biotechnol. 28, 1327–1350. doi: 10.1007/s11274-011-0979-9, 22805914

[ref20] Blagodatskaya E. Kuzyakov Y. (2013). Active microorganisms in soil: critical review of estimation criteria and approaches. Soil Biol. Biochem. 67, 192–211. doi: 10.1016/j.soilbio.2013.08.024.

[ref21] Blair P. M. Land M. L. Piatek M. J. Jacobson D. A. Lu T.-Y. S. Doktycz M. J. . (2018). Exploration of the biosynthetic potential of the Populus microbiome. MSystems. 3:e00045-18. doi: 10.1128/mSystems.00045-18 30320216 PMC6172771

[ref22] Bokulich N. Dillon M. Bolyen E. Kaehler B. Huttley G. Caporaso J. (2018). q2-sample-classifier: machine-learning tools for microbiome classification and regression. J. Open Source Softw. 3:934. doi: 10.21105/joss.00934, 31552137 PMC6759219

[ref23] Bolyen E. Rideout J. R. Dillon M. R. Bokulich N. A. Abnet C. C. Al-Ghalith G. A. . (2019). Reproducible, interactive, scalable and extensible microbiome data science using QIIME 2. Nat. Biotechnol. 37, 852–857. doi: 10.1038/s41587-019-0209-9, 31341288 PMC7015180

[ref24] Brunner-Mendoza C. Reyes-Montes M. d. R. Moonjely S. Bidochka M. J. Toriello C. (2019). A review on the genus Metarhizium as an entomopathogenic microbial biocontrol agent with emphasis on its use and utility in Mexico. Biocontrol Sci. Tech. 29, 83–102. doi: 10.1080/09583157.2018.1531111

[ref25] Burns R. G. DeForest J. L. Marxsen J. Sinsabaugh R. L. Stromberger M. E. Wallenstein M. D. . (2013). Soil enzymes in a changing environment: current knowledge and future directions. Soil Biol. Biochem. 58, 216–234. doi: 10.1016/j.soilbio.2012.11.009

[ref26] Cavicchioli R. Ripple W. J. Timmis K. N. Azam F. Bakken L. R. Baylis M. . (2019). Scientists’ warning to humanity: microorganisms and climate change. Nat. Rev. Microbiol. 17, 569–586. doi: 10.1038/s41579-019-0222-5, 31213707 PMC7136171

[ref27] Cesari A. Paulucci N. Lopez-Gomez M. Hidalgo-Castellanos J. Pla C. L. Dardanelli M. S. (2019). Restrictive water condition modifies the root exudates composition during peanut-PGPR interaction and conditions early events, reversing the negative effects on plant growth. Plant Physiol. Biochem. 142, 519–527. doi: 10.1016/j.plaphy.2019.08.015, 31450055

[ref28] Chai R. Ye X. Ma C. Wang Q. Tu R. Zhang L. . (2019). Greenhouse gas emissions from synthetic nitrogen manufacture and fertilization for main upland crops in China. Carbon Balance Manag. 14:20. doi: 10.1186/s13021-019-0133-9, 31889246 PMC7227229

[ref29] Chang H.-X. Haudenshield J. S. Bowen C. R. Hartman G. L. (2017). Metagenome-wide association study and machine learning prediction of bulk soil microbiome and crop productivity. Front. Microbiol. 8:519. doi: 10.3389/fmicb.2017.00519, 28421041 PMC5378059

[ref30] Chávez-Ramírez B. Kerber-Díaz J. C. Acoltzi-Conde M. C. Ibarra J. A. Vásquez-Murrieta M. S. de Estrada-los Santos P. (2020). Inhibition of rhizoctonia solani RhCh-14 and Pythium ultimum PyFr-14 by Paenibacillus polymyxa NMA1017 and Burkholderia cenocepacia CACua-24: a proposal for biocontrol of phytopathogenic fungi. Microbiol. Res. 230:126347. doi: 10.1016/j.micres.2019.126347, 31586859

[ref31] Chen Y. Li S. Liu N. He H. Cao X. Lv C. . (2021). Effects of different types of microbial inoculants on available nitrogen and phosphorus, soil microbial community, and wheat growth in high-P soil. Environ. Sci. Pollut. Res. 28, 23036–23047. doi: 10.1007/s11356-020-12203-y, 33438124

[ref32] Chen L. Wu Y. D. Chong X. Y. Xin Q. H. Wang D. X. Bian K. (2020). Seed-borne endophytic Bacillus velezensis LHSB1 mediate the biocontrol of peanut stem rot caused by sclerotium rolfsii. J. Appl. Microbiol. 128, 803–813. doi: 10.1111/jam.14508, 31705716

[ref33] Chu H. Gao G.-F. Ma Y. Fan K. Delgado-Baquerizo M. (2020). Soil microbial biogeography in a changing world: recent advances and future perspectives. mSystems 5:e00803-19. doi: 10.1128/mSystems.00803-19. 32317392 PMC7174637

[ref34] Coban O. De Deyn G. B. van der Ploeg M. (2022). Soil microbiota as game-changers in restoration of degraded lands. Science 375:990. doi: 10.1126/science.abe0725, 35239372

[ref35] Cole J. R. Wang Q. Fish J. A. Chai B. McGarrell D. M. Sun Y. . (2014). Ribosomal database project: data and tools for high throughput rRNA analysis. Nucleic Acids Res. 42, D633–D642. doi: 10.1093/nar/gkt1244, 24288368 PMC3965039

[ref36] Coleman-Derr D. Tringe S. G. (2014). Building the crops of tomorrow: advantages of symbiont-based approaches to improving abiotic stress tolerance. Front. Microbiol. 5, 1–6. doi: 10.3389/fmicb.2014.00283, 24936202 PMC4047557

[ref37] Compant S. Duffy B. Nowak J. Clément C. Barka E. A. (2005). Use of plant growth-promoting bacteria for biocontrol of plant diseases: principles, mechanisms of action, and future prospects. Appl. Environ. Microbiol. 71, 4951–4959. doi: 10.1128/AEM.71.9.4951-4959.2005, 16151072 PMC1214602

[ref38] Compant S. Samad A. Faist H. Sessitsch A. (2019). A review on the plant microbiome: ecology, functions, and emerging trends in microbial application. J. Adv. Res. 19, 29–37. doi: 10.1016/j.jare.2019.03.004, 31341667 PMC6630030

[ref39] Cordovez V. Dini-Andreote F. Carrión V. J. Raaijmakers J. M. (2019). Ecology and evolution of plant microbiomes. Ann. Rev. Microbiol. 73, 69–88. doi: 10.1146/annurev-micro-090817-062524, 31091418

[ref40] Dal Cortivo C. Ferrari M. Visioli G. Lauro M. Fornasier F. Barion G. . (2020). Effects of seed-applied biofertilizers on rhizosphere biodiversity and growth of common wheat (Triticum aestivum L.) in the field. Front. Plant Sci. 11:72. doi: 10.3389/fpls.2020.00072, 32174929 PMC7054350

[ref41] Dębska B. Długosz J. Piotrowska-Długosz A. Banach-Szott M. (2016). The impact of a bio-fertilizer on the soil organic matter status and carbon sequestration – results from a field-scale study. J. Soils Sediments 16, 2335–2343. doi: 10.1007/s11368-016-1430-5

[ref42] Delgado-Baquerizo M. Maestre F. T. Reich P. B. Trivedi P. Osanai Y. Liu Y. R. . (2016). Carbon content and climate variability drive global soil bacterial diversity patterns. Ecol. Monogr. 86, 373–380. doi: 10.1002/ecm.1216.

[ref43] Delgado-Baquerizo M. Oliverio A. M. Brewer T. E. Benavent-González A. Eldridge D. J. Bardgett R. D. . (2018). A global atlas of the dominant bacteria across soil biomes. Science 359, 320–325. doi: 10.1126/science.aap9516, 29348236

[ref44] Demilie W. B. (2024). Plant disease detection and classification techniques: a comparative study of the performances. J. Big Data 11:5. doi: 10.1186/s40537-023-00863-9

[ref45] Dhouib H. Zouari I. Ben Abdallah D. Belbahri L. Taktak W. Triki M. A. . (2019). Potential of a novel endophytic *Bacillus velezensis* in tomato growth promotion and protection against verticillium wilt disease. Biol. Control 139:104092. doi: 10.1016/j.biocontrol.2019.104092

[ref5001] Dong Q. Liu Q. Goodwin P. H. Deng X. Xu W. Xia M. . (2023). Isolation and Genome-Based Characterization of Biocontrol Potential of Bacillus siamensis YB-1631 against Wheat Crown Rot Caused by Fusarium pseudograminearum. J Fungi (Basel). 9:547. doi: 10.3390/jof9050547 37233258 PMC10219336

[ref46] Du Y. Han X. Tsuda K. (2025). Microbiome-mediated plant disease resistance: recent advances and future directions. J. Gen. Plant Pathol. 91, 1–17. doi: 10.1007/s10327-024-01204-1

[ref47] Dubey A. Malla A. M. Khan F. Chowdhary K. Yadav S. Kumar A. . (2019). Soil microbiome: a key player for conservation of soil health under changing climate. Biodivers. Conserv. 28, 2405–2429. doi: 10.1007/s10531-019-01760-5

[ref48] Duke K. A. Becker M. G. Girard I. J. Millar J. L. Dilantha Fernando W. G. Belmonte M. F. . (2017). The biocontrol agent *Pseudomonas chlororaphis* PA23 primes *Brassica napus* defenses through distinct gene networks. BMC Genomics 18:467. doi: 10.1186/s12864-017-3848-6, 28629321 PMC5477169

[ref49] Dutta J. Bora U. (2019). “Role of PGPR for alleviating Aluminum toxicity in acidic soil” in Plant growth promoting rhizobacteria for sustainable stress management: volume 1: rhizobacteria in abiotic stress management. eds. Sayyed R. Z. Arora N. K. Reddy M. S. (Singapore: Springer), 309–326.

[ref50] Edgar R. C. (2010). Search and clustering orders of magnitude faster than BLAST. Bioinformatics 26, 2460–2461. doi: 10.1093/bioinformatics/btq461, 20709691

[ref51] Egamberdieva D. Wirth S. Jabborova D. Räsänen L. A. Liao H. (2017). Coordination between Bradyrhizobium and Pseudomonas alleviates salt stress in soybean through altering root system architecture. J. Plant Interact. 12, 100–107. doi: 10.1080/17429145.2017.1294212

[ref52] Elad Y. (2000). Biological control of foliar pathogens by means of *Trichoderma harzianum* and potential modes of action. Crop Prot. 19, 709–714. doi: 10.1016/S0261-2194(00)00094-6

[ref53] Elad Y. David D.R.R. Levi T. Kapat A. Kirshner B. Guvrin E. (1999). *Trichoderma harzianum* T39 – mechanisms of biocontrol of foliar pathogens. In Modern Fungicides and Antifungal Compounds II 12th International Reinhardsbrunn Symposium, 459–467, Friedrichroda, Thuringia, Germany.

[ref54] Elazzazy A. M. Baeshen M. N. Alasmi K. M. Alqurashi S. I. Desouky S. E. Khattab S. M. R. (2025). Where biology meets engineering: scaling up microbial nutraceuticals to bridge nutrition, therapeutics, and global impact. Microorganisms 13:566. doi: 10.3390/microorganisms13030566, 40142459 PMC11945976

[ref55] Elshahat M. R. Ahmed A. A. Enas A. H. Fekria M. S. (2016). Plant growth promoting rhizobacteria and their potential for biocontrol of phytopathogens. Afr. J. Microbiol. Res. 10, 486–504. doi: 10.5897/ajmr2015.7714

[ref56] EU Communication: EU biodiversity strategy for 2030 – bringing nature back into our liveso title. (2019). Available online at: https://ec.europa.eu/info/strategy/priorities-2019-2024/european-green-deal/actions-being-taken-eu/eu-biodiversity-strategy-2030_en#documents (Accessed January 31, 2025).

[ref57] European Commission. (2020). FOOD 2030 pathways for action. The microbiome world: a life science opportunity for our society and our planet. Available online at: https://op.europa.eu/en/publication-detail/-/publication/dd1ca03d-2564-11eb-9d7e-01aa75ed71a1/language-en

[ref58] Fan Q. Wang H. Mao C. Li J. Zhang X. Grenier D. . (2022). Structure and signal regulation mechanism of interspecies and interkingdom quorum sensing system receptors. J. Agric. Food Chem. 70, 429–445. doi: 10.1021/acs.jafc.1c04751, 34989570

[ref59] Ferrarezi J. A. Defant H. de Souza L. F. Azevedo J. L. Hungria M. Quecine M. C. (2023). Meta-omics integration approach reveals the effect of soil native microbiome diversity in the performance of inoculant *Azospirillum brasilense*. Front. Plant Sci. 14:1172839. doi: 10.3389/fpls.2023.1172839, 37457347 PMC10340089

[ref60] Fierer N. (2017). Embracing the unknown: disentangling the complexities of the soil microbiome. Nat. Rev. Microbiol. 15, 579–590. doi: 10.1038/nrmicro.2017.87, 28824177

[ref61] Fierer N. Jackson R. B. (2006). The diversity and biogeography of soil bacterial communities. Proc. Natl. Acad. Sci. USA 103, 626–631. doi: 10.1073/pnas.0507535103, 16407148 PMC1334650

[ref62] Figueiredo M. Mergulhão A. Kuklinsky Sobral J. Lira Junior M. Araujo A. (2013). “Chapter 10: biological nitrogen fixation: importance, associated diversity, and estimates” in Plant microbe symbiosis: fundamentals and advances, (ed.) Arora NK, New Delhi, Heidelberg, New York, Dordrecht, London: Springer. 1–459. doi: 10.1007/978-81-322-1287-4

[ref63] Fikri A. S. I. Rahman I. A. Nor N. S. M. Hamzah A. (2018). Isolation and identification of local bacteria endophyte and screening of its antimicrobial property against pathogenic bacteria and fungi. AIP Conf. Proc. 1940:020072. doi: 10.1063/1.5027987

[ref64] Filker S. Sommaruga R. Vila I. Stoeck T. (2016). Microbial eukaryote plankton communities of high-mountain lakes from three continents exhibit strong biogeographic patterns. Mol. Ecol. 25, 2286–2301. doi: 10.1111/mec.13633, 27029537 PMC4976798

[ref65] Fira D. Dimkić I. Berić T. Lozo J. Stanković S. (2018). Biological control of plant pathogens by *Bacillus* species. J. Biotechnol. 285, 44–55. doi: 10.1016/j.jbiotec.2018.07.044, 30172784

[ref66] Fitzpatrick C. R. Mustafa Z. Viliunas J. (2019). Soil microbes alter plant fitness under competition and drought. J. Evolut. Biol. 32, 438–450. doi: 10.1111/jeb.13426, 30739360

[ref68] Food and Agriculture Organization of the United Nations [FAO] and ITPS, (2021). Recarbonizing global soils – a technical manual of recommended sustainable soil management. Volume 3: Cropland, grassland, integrated systems, and farming approaches – practices overview.

[ref69] Frąc M. Hannula S. E. Belka M. Jędryczka M. (2018). Fungal biodiversity and their role in soil health. Front. Microbiol. 9:707. doi: 10.3389/fmicb.2018.00707, 29755421 PMC5932366

[ref70] Frąc M. Hannula E. S. Bełka M. Salles J. F. Jedryczka M. (2022). Soil mycobiome in sustainable agriculture. Front. Microbiol. 13:1033824. doi: 10.3389/fmicb.2022.1033824, 36519160 PMC9742577

[ref71] Franco Lucas B. Costa J. A. V. Brunner T. A. (2021). Superfoods: drivers for consumption. J. Food Prod. Mark. 27, 1–9. doi: 10.1080/10454446.2020.1869133

[ref72] Franzosa E. A. Hsu T. Sirota-Madi A. Shafquat A. Abu-Ali G. Morgan X. C. . (2015). Sequencing and beyond: integrating molecular “omics” for microbial community profiling. Nat. Rev. Microbiol. 13, 360–372. doi: 10.1038/nrmicro3451, 25915636 PMC4800835

[ref73] German D. P. Marcelo K. R. B. Stone M. M. Allison S. D. (2012). The Michaelis–Menten kinetics of soil extracellular enzymes in response to temperature: a cross-latitudinal study. Glob. Change Biol. 18, 1468–1479. doi: 10.1111/j.1365-2486.2011.02615.x

[ref74] Goodwin S. McPherson J. D. McCombie W. R. (2016). Coming of age: ten years of next-generation sequencing technologies. Nat. Rev. Genet. 17, 333–351. doi: 10.1038/nrg.2016.49, 27184599 PMC10373632

[ref75] Grzyb Z. S. Paszt L. S. Piotrowski W. Malusa E. (2015). The influence of mycorrhizal fungi on the growth of apple and sour cherry maidens fertilized with different bioproducts in the organic nursery. J. Life Sci. 9, 221–228. doi: 10.17265/1934-7391/2015.05.005

[ref76] Gu S. Zhou X. Yu H. Yan H. Wang Y. Liu Y. . (2023). Microbial and chemical fertilizers for restoring degraded alpine grassland. Biol. Fertil. Soils 59, 911–926. doi: 10.1007/s00374-023-01759-9

[ref77] Hacquard S. Garrido-Oter R. González A. Spaepen S. Ackermann G. Lebeis S. . (2015). Microbiota and host nutrition across plant and animal kingdoms. Cell Host Microbe 17, 603–616. doi: 10.1016/j.chom.2015.04.009, 25974302

[ref78] Han J. H. Shim H. Shin J. H. Kim K. S. (2015). Antagonistic activities of Bacillus spp. strains isolated from tidal flat sediment towards anthracnose pathogens *Colletotrichum acutatum* and *C. gloeosporioides* in South Korea. Plant Pathol. J. 31, 165–175. doi: 10.5423/PPJ.OA.03.2015.0036, 26060435 PMC4453997

[ref79] Haran S. Schickler H. Oppenheim A. Chet I. (1996). Differential expression of *Trichoderma harzianum* chitinases during mycoparasitism. Phytopathology 86, 980–985.

[ref80] Harel Y. M. Kolton M. Elad Y. Rav-david D. Cytryn E. Borenstein M. . (2011). Induced systemic resistance in strawberry (Fragaria × ananassa) to powdery mildew using various control agents. IOBC Bull 71, 47–51.

[ref81] Hartman K. van der Heijden M. G. A. Wittwer R. A. Banerjee S. Walser J. C. Schlaeppi K. (2018). Cropping practices manipulate abundance patterns of root and soil microbiome members paving the way to smart farming. Microbiome 6:114. doi: 10.1186/s40168-017-0389-9, 29338764 PMC5771023

[ref82] Hartmann M. Widmer F. (2006). Community structure analyses are more sensitive to differences in soil bacterial communities than anonymous diversity indices. Appl. Environ. Microbiol. 72, 7804–7812. doi: 10.1128/AEM.01464-06, 17041161 PMC1694274

[ref83] Hassani M. A. Durán P. Hacquard S. (2018). Microbial interactions within the plant holobiont. Microbiome 6, 1–17. doi: 10.1186/s40168-018-0445-0, 29587885 PMC5870681

[ref84] Hellweger F. L. van Sebille E. Fredrick N. D. (2014). Biogeographic patterns in ocean microbes emerge in a neutral agent-based model. Science 345, 1346–1349. doi: 10.1126/science.1254421, 25214628

[ref85] Heuer H. Smalla K. (2012). Plasmids foster diversification and adaptation of bacterial populations in soil. FEMS Microbiol. Rev. 36, 1083–1104. doi: 10.1111/j.1574-6976.2012.00337.x, 22393901

[ref86] Hirsch P. R. Mauchline T. H. (2012). Who’s who in the plant root microbiome? Nature Biotechnol. 30, 961–962. doi: 10.1038/nbt.2387, 23051815

[ref87] Hong S. Bunge J. Leslin C. Jeon S. Epstein S. S. (2009). Polymerase chain reaction primers miss half of rRNA microbial diversity. ISME J. 3, 1365–1373. doi: 10.1038/ismej.2009.89, 19693101

[ref88] Hu C. Xia X. Chen Y. Han X. (2018). Soil carbon and nitrogen sequestration and crop growth as influenced by long-term application of effective microorganism compost. Chil. J. Agric. Res. 78, 13–22. doi: 10.4067/S0718-58392018000100013

[ref89] Hugenholtz P. Pace N. (1996). Identifying microbial diversity in the natural environment: a molecular phylogenetic approach. Trends Biotechnol. 14, 190–197. doi: 10.1016/0167-7799(96)10025-1, 8663938

[ref90] Jacoby R. Peukert M. Succurro A. Koprivova A. Kopriva S. (2017). The role of soil microorganisms in plant mineral nutrition—current knowledge and future directions. Front. Plant Sci. 8:1617. doi: 10.3389/fpls.2017.01617, 28974956 PMC5610682

[ref91] Jansson J. K. Baker E. S. (2016). A multi-omic future for microbiome studies. Nat. Microbiol. 1:16049. doi: 10.1038/nmicrobiol.2016.49, 27572648

[ref92] Jansson J. K. Hofmockel K. S. (2020). Soil microbiomes and climate change. Nat. Rev. Microbiol. 18:35. doi: 10.1038/s41579-019-0265-7, 31586158

[ref93] Ji S. An Y. B. Zhang H. Wang Y. Liu Z. (2021). Trichoderma biofertilizer (mixTroTha) mediates Malus sieversii resistance to Alternaria alternata. Biol. Control 156:104539. doi: 10.1016/j.biocontrol.2021.104539

[ref94] Kandasamy S. Liu E. Y. R. Patterson G. Saldias S. Ali S. Lazarovits G. (2019). Introducing key microbes from high productive soil transforms native soil microbial community of low productive soil. Microbiology 8:e895. doi: 10.1002/mbo3.895, 31250991 PMC6813456

[ref95] Kantachote D. Nunkaew T. Kantha T. Chaiprapat S. (2016). Biofertilizers from *Rhodopseudomonas palustris* strains to enhance rice yields and reduce methane emissions. Appl. Soil Ecol. 100, 154–161. doi: 10.1016/j.apsoil.2015.12.015

[ref96] Kaushal M. Wani S. P. (2016). Plant-growth-promoting rhizobacteria: drought stress alleviators to ameliorate crop production in drylands. Ann. Microbiol. 66, 35–42. doi: 10.1007/s13213-015-1112-3

[ref97] Kaushik P. Sandhu O.S. Brar N.S. Kumar V. Malhi G.S. Kesh H. . (2020) Soil metagenomics: prospects and challenges. (ed.) Radhakrishnan R.. London: Mycorrhizal Fungi - Utilization in Agriculture and Forestry, IntechOpen. doi: 10.5772/intechopen.93306

[ref98] Kim Y. S. Lee Y. Cheon W. Park J. Kwon H. T. Balaraju K. . (2021). Characterization of *Bacillus velezensis* AK-0 as a biocontrol agent against apple bitter rot caused by *Colletotrichum gloeosporioides*. Sci. Rep. 11:626. doi: 10.1038/s41598-020-80231-2, 33436839 PMC7804190

[ref99] Knight R. Vrbanac A. Taylor B. C. Aksenov A. Callewaert C. Debelius J. . (2018). Best practices for analysing microbiomes. Nat. Rev. Microbiol. 16, 410–422. doi: 10.1038/s41579-018-0029-9, 29795328

[ref100] Köhl J. Kolnaar R. Ravensberg W. J. (2019). Mode of action of microbial biological control agents against plant diseases: relevance beyond efficacy. Front. Plant Sci. 10:845. doi: 10.3389/fpls.2019.00845, 31379891 PMC6658832

[ref101] Krey T. Caus M. Baum C. Ruppel S. Eichler-Löbermann B. (2011). Interactive effects of plant growth–promoting rhizobacteria and organic fertilization on P nutrition of *Zea mays* L. and *Brassica napus* L. J. Plant Nutr. Soil Sci. 174, 602–613. doi: 10.1002/jpln.200900349

[ref102] Kuan K. B. Othman R. Rahim K. A. Shamsuddin Z. H. (2016). Plant growth-promoting rhizobacteria inoculation to enhance vegetative growth, nitrogen fixation and nitrogen remobilisation of maize under greenhouse conditions. PLoS One 11:e0152478. doi: 10.1371/journal.pone.0152478, 27011317 PMC4807084

[ref103] Kulak M. Graves A. Chatterton J. (2013). Reducing greenhouse gas emissions with urban agriculture: a life cycle assessment perspective. Landsc. Urban Plan. 111, 68–78. doi: 10.1016/j.landurbplan.2012.11.007.

[ref104] Kumar S. Meena R. S. Singh R. K. Munir T. M. Datta R. Danish S. . (2021). Soil microbial and nutrient dynamics under different sowings environment of Indian mustard (*Brassica juncea* L.) in rice based cropping system. Sci. Rep. 11:5289. doi: 10.1038/s41598-021-84742-4, 33674666 PMC7935972

[ref105] Kuntz M.G.F. Fernandes R. do Rosário V.A. Fiates G.M.R. de Moraes Trinda E.B.S. (2015) Inulin: Modulation of intestinal microbiota and propects in new products. Available online at: https://www.researchgate.net/publication/296951803 (Accessed January 31, 2025).

[ref106] Larsson D. Flach C.-F. (2021). Antibiotic resistance in the environment. Nat. Rev. Microbiol. 202, 1–13. doi: 10.1038/s41579-021-00649-x PMC8567979 34737424

[ref107] Leach J. E. Triplett L. R. Argueso C. T. Trivedi P. (2017). Communication in the Phytobiome. Cell 169, 587–596. doi: 10.1016/j.cell.2017.04.025, 28475891

[ref108] Leite M. F. A. van den Broek S. W. E. B. Kuramae E. E. (2022). Current challenges and pitfalls in soil metagenomics. Microorganisms 10:1900. doi: 10.3390/microorganisms10101900, 36296177 PMC9606969

[ref109] Levy A. Salas González I. Mittelviefhaus M. Clingenpeel S. Herrera Paredes S. Miao J. . (2018). Genomic features of bacterial adaptation to plants. Nat. Genet. 50, 138–150. doi: 10.1038/s41588-017-0012-9, 29255260 PMC5957079

[ref110] Li S. Xie D. Ge X. Dong W. Luan J. (2022). Altered diversity and functioning of soil and root-associated microbiomes by an invasive native plant. Plant Soil 480, 1–16. doi: 10.1007/s11104-022-05338-z

[ref111] Lipa P. Kozieł M. Janczarek M. (2017). Zjawisko quorum sensing bakterii gram-ujemnych: cząsteczki sygnałowe i inhibitory oraz ich potencjalne zastosowanie terapeutyczne [quorum sensing in gram-negative bacteria: signal molecules, inhibitors and their potential therapeutic application]. Postepy Biochem. 63, 242–260. 29374426

[ref112] Liu X. Le Roux X. Falcao-Salles J. (2022). The legacy of microbial inoculants in agroecosystems and potential for tackling climate change challenges. iScience 25:103821. doi: 10.1016/j.isci.2022.103821, 35243218 PMC8867051

[ref113] Liu Q. Pang Z. Yang Z. Nyumah F. Hu C. Lin W. . (2021). Bio-fertilizer affects structural dynamics, function, and network patterns of the sugarcane Rhizospheric microbiota. Microb. Ecol. 84, 1195–1211. doi: 10.1007/s00248-021-01932-3, 34820729 PMC9747866

[ref114] Lombard N. Prestat E. van Elsas J. D. Simonet P. (2011). Soil-specific limitations for access and analysis of soil microbial communities by metagenomics. FEMS Microb. Ecol. 78, 31–49. doi: 10.1111/j.1574-6941.2011.01140.x, 21631545

[ref115] Loreau M. Naeem S. Inchausti P. Bengtsson J. Grime J. P. Hector A. . (2001). Biodiversity and ecosystem functioning: current knowledge and future challenges. Science 294, 804–808. doi: 10.1126/science.1064088, 11679658

[ref116] Lorito M. Farkas V. Rebuffat S. Bodo B. Kubicek C. P. (1996). Cell Wall synthesis is a major target of Mycoparasitic antagonism by Trichoderma harzianum. J. Bacteriol. 178, 6382–6385. doi: 10.1128/jb.178.21.6382-6385.1996, 8892847 PMC178518

[ref117] Luo G. Xue C. Jiang Q. Xiao Y. Zhang F. Guo S. . (2020). Soil carbon, nitrogen, and phosphorus cycling microbial populations and their resistance to global change depend on soil C:N:P stoichiometry. mSystems 5:e00162-20. doi: 10.1128/msystems.00162-20, 32606023 PMC7329320

[ref118] Mącik M. Gryta A. Frąc M. (2020a). Biofertilizers in agriculture: an overview on concepts, strategies and effects on soil microorganisms. Adv. Agron. 162, 31–87. doi: 10.1016/bs.agron.2020.02.001

[ref119] Mącik M. Gryta A. Sas-Paszt L. Frąc M. (2020b). The status of soil microbiome as affected by the application of phosphorus biofertilizer: fertilizer enriched with beneficial bacterial strains. Int. J. Mol. Sci. 21:8003. doi: 10.3390/ijms21218003, 33121206 PMC7663420

[ref120] Mącik M. Gryta A. Sas-Paszt L. Frąc M. (2022). Composition, activity and diversity of bacterial and fungal communities responses to inputs of phosphorus fertilizer enriched with beneficial microbes in degraded Brunic Arenosol. Land Degrad. Dev. 33, 844–865. doi: 10.1002/ldr.4179

[ref121] Madhaiyan M. Poonguzhali S. Kang B.-G. Lee Y.-J. Chung J.-B. Sa T.-M. (2010). Effect of co-inoculation of methylotrophic *Methylobacterium oryzae* with *Azospirillum brasilense* and *Burkholderia pyrrocinia* on the growth and nutrient uptake of tomato, red pepper and rice. Plant Soil 328, 71–82. doi: 10.1007/s11104-009-0083-1

[ref122] Mahmud A. A. Upadhyay S. K. Srivastava A. K. Bhojiya A. A. (2021). Biofertilizers: a nexus between soil fertility and crop productivity under abiotic stress. Curr. Res. Environ. Sustain. 3:100063. doi: 10.1016/j.crsust.2021.100063

[ref123] Mandels M. Andreotti R. (1978). Problems and challenges in the cellulose to cellulase fermentation. Process Biochem. 13:5.

[ref124] Mao T. Jiang X. (2021). Changes in microbial community and enzyme activity in soil under continuous pepper cropping in response to *Trichoderma hamatum* MHT1134 application. Sci. Rep. 11:21585. doi: 10.1038/s41598-021-00951-x, 34732764 PMC8566488

[ref125] Marchesi J. R. Ravel J. (2015). The vocabulary of microbiome research: a proposal. Microbiome 3:31. doi: 10.1186/s40168-015-0094-5, 26229597 PMC4520061

[ref126] Maron P.-A. Sarr A. Kaisermann A. Lévêque J. Mathieu O. Guigue J. . (2018). High microbial diversity promotes soil ecosystem functioning. Appl. Environ. Microbiol. 84:e02738. doi: 10.1128/AEM.02738-17, 29453268 PMC5930326

[ref127] Masuda Y. Mise K. Xu Z. Zhang Z. Shiratori Y. Senoo K. . (2024). Global soil metagenomics reveals distribution and predominance of Deltaproteobacteria in nitrogen-fixing microbiome. Microbiome 12:95. doi: 10.1186/s40168-024-01812-1, 38790049 PMC11127431

[ref128] Mawarda P. C. Le Roux X. van Dirk Elsas J. Salles J. F. (2020). Deliberate introduction of invisible invaders: a critical appraisal of the impact of microbial inoculants on soil microbial communities. Soil Biol. Biochem. 148:107874. doi: 10.1016/j.soilbio.2020.107874

[ref129] Mesny F. Hacquard S. Thomma B. P. (2023). Co-evolution within the plant holobiont drives host performance. EMBO Rep. 24:e57455. doi: 10.15252/embr.202357455, 37471099 PMC10481671

[ref130] Mitter B. Pfaffenbichler N. Flavell R. Compant S. Antonielli L. Petric A. . (2017). A new approach to modify plant microbiomes and traits by introducing beneficial bacteria at flowering into progeny seeds. Front. Microbiol. 8:11. doi: 10.3389/fmicb.2017.00011, 28167932 PMC5253360

[ref131] Mitter E. K. Tosi M. Obregón D. Dunfield K. E. Germida J. J. (2021). Rethinking crop nutrition in times of modern microbiology: innovative biofertilizer technologies. Front. Sustain. Food Syst. 5:606815. doi: 10.3389/fsufs.2021.606815

[ref132] Mohanty P. Singh P. K. Chakraborty D. Mishra S. Pattnaik R. (2021). Insight into the role of PGPR in sustainable agriculture and environment. Front. Sustain. Food Syst. 5, 1–12. doi: 10.3389/fsufs.2021.667150

[ref133] Müller D. B. Vogel C. Bai Y. Vorholt J. A. (2016). The plant microbiota: systems-level insights and perspectives. Annu. Rev. Genet. 50, 211–234. doi: 10.1146/annurev-genet-120215-034952, 27648643

[ref134] Mumtaz M. Z. Castellane T. C. L. Shahid I. Tamburini E. Bouizgarne B. (2025). Plant mineral–microbe interactions: harnessing synergistic potential for sustainable agriculture. Front. Microbiol. 16:1677458. doi: 10.3389/fmicb.2025.1677458, 41078520 PMC12509987

[ref135] Muñoz C. Y. Zhou L. Yi Y. Kuipers O. P. (2021). Genome mining of antimicrobial gene clusters from phyllospheric bacteria isolated from *Solanum lycopersicum* and *Lactuca sativa* to identify biocontrol properties. doi: 10.21203/rs.3.rs-633887/v1 PMC8862347 35189837

[ref136] Nannipieri P. Trasar-Cepeda C. Dick R. P. (2018). Soil enzyme activity: a brief history and biochemistry as a basis for appropriate interpretations and meta-analysis. Biol. Fertil. Soils 54, 11–19. doi: 10.1007/s00374-017-1245-6

[ref137] Niu X. Song L. Xiao Y. Ge W. (2018). Drought-tolerant plant growth-promoting rhizobacteria associated with foxtail millet in a semi-arid agroecosystem and their potential in alleviating drought stress. Front. Microbiol. 8:2580. doi: 10.3389/fmicb.2018.02580, 29379471 PMC5771373

[ref138] Nosheen A. Yasmin H. Naz R. Bano A. Keyani R. Hussain I. (2018). *Pseudomonas putida* improved soil enzyme activity and growth of kasumbha under low input of mineral fertilizers. Soil Sci. Plant Nutr. 64, 520–525. doi: 10.1080/00380768.2018.1461002

[ref139] O’Brien P. A. (2017). Biological control of plant diseases. Australas. Plant Pathol. 46, 293–304. doi: 10.1007/s13313-017-0481-4

[ref140] Oszust K. Cybulska J. Frąc M. (2020). How do Trichoderma genus Fungi win a nutritional competition Battle against soft fruit pathogens? A report on niche overlap nutritional potentiates. Int. J. Mol. Sci. 21:4235. doi: 10.3390/ijms21124235, 32545883 PMC7352470

[ref141] Oszust K. Frąc M. (2021). First report on the microbial communities of the wild and planted raspberry rhizosphere – a statement on the taxa, processes and a new indicator of functional diversity. Ecol. Indic. 121:107117. doi: 10.1016/j.ecolind.2020.107117

[ref142] Oszust K. Pylak M. Frąc M. (2021). Trichoderma-based biopreparation with prebiotics supplementation for the naturalization of raspberry plant rhizosphere. Int. J. Mol. Sci. 22:6356. doi: 10.3390/ijms 34198606 PMC8232080

[ref143] Panek J. Frąc M. Treder K. Pawłowska A. Michałowska D. Vink S.N. . (2021). Mycobiome composition of rhizosphere of selected potato cultivars. Metagenomes of various environment, Symposium, Warsaw, Poland,

[ref144] Pang Z. Dong F. Liu Q. Lin W. Hu C. Yuan Z. (2021). Soil metagenomics reveals effects of continuous sugarcane cropping on the structure and functional pathway of Rhizospheric microbial community. Front. Microbiol. 12:627569. doi: 10.3389/fmicb.2021.627569, 33746921 PMC7973049

[ref145] Payne A. Holmes N. Rakyan V. Loose M. (2019). BulkVis: a graphical viewer for Oxford nanopore bulk FAST5 files. Bioinformatics 35, 2193–2198. doi: 10.1093/bioinformatics/bty841, 30462145 PMC6596899

[ref146] Pedregosa F. Varoquaux G. Gramfort A. Michel V. Thirion B. Grisel O. . (2011). Scikit-learn: machine learning in Python. J. Mach. Learn. Res. 12, 2825–2830.

[ref147] Pellegrini M. Teixeira Filho M. C. M. Panneerselvam P. (2025). Microbial-based inoculants for agriculture: production and improvement of commercial formulations. Front. Ind. Microbiol. 3:1664174. doi: 10.3389/finmi.2025.1664174

[ref148] Pertile G. Lamorski K. Bieganowski A. Boguta P. Brzezińska M. Polakowski C. . (2021). Immediate effects of the application of various fungal strains with urea fertiliser on microbiome structure and functions and their relationships with the physicochemical parameters of two different soil types. Appl. Soil Ecol. 163:103972. doi: 10.1016/j.apsoil.2021.103972

[ref149] Pieterse C. M. Zamioudis C. Berendsen R. L. Weller D. M. Van Wees S. C. Bakker P. A. (2014). Induced systemic resistance by beneficial microbes. Annu. Rev. Phytopathol. 52, 347–375. doi: 10.1146/annurev-phyto-082712-102340, 24906124

[ref150] Pokluda R. Ragasová L. Jurica M. Kalisz A. Komorowska M. Niemiec M. . (2021). Effects of growth promoting microorganisms on tomato seedlings growing in different media conditions. PLoS One 16:e0259380. doi: 10.1371/journal.pone.0259380, 34731216 PMC8565787

[ref151] Poonam P. K. (2021). Plant growth promoting rhizobacteria (PGPR): a review. Int. J. Curr. Microbiol. Appl. Sci. 10, 882–886. doi: 10.20546/ijcmas.2021.1004.093

[ref152] Prasad R. Kumar M. Varma A. (2015). “Role of PGPR in soil fertility and plant health” in Plant-growth-promoting rhizobacteria (PGPR) and medicinal plants. eds. Egamberdieva D. Shrivastava S. Varma A. (Cham: Springer International Publishing), 247–260.

[ref153] Prosser J. I. (2015). Dispersing misconceptions and identifying opportunities for the use of ‘omics’ in soil microbial ecology. Nat. Rev. Microbiol. 13, 439–446. doi: 10.1038/nrmicro3468, 26052662

[ref154] Purahong W. Mapook A. Wu Y.-T. Chen C.-T. (2019). Characterization of the Castanopsis carlesii deadwood mycobiome by Pacbio sequencing of the full-length fungal nuclear ribosomal internal transcribed spacer (ITS). Front. Microbiol. 10. doi: 10.3389/fmicb.2019.00983, 31191462 PMC6540943

[ref155] Pylak M. Oszust K. Frąc M. (2019). Review report on the role of bioproducts, biopreparations, biostimulants and microbial inoculants in organic production of fruit. Rev. Environ. Sci. Biotechnol. 18, 597–616. doi: 10.1007/s11157-019-09500-5

[ref156] Pylak M. Oszust K. Frąc M. (2021). Optimization of growing medium and preservation methods for plant beneficial bacteria, and formulating a microbial biopreparation for raspberry naturalization. Agronomy 11:2521. doi: 10.3390/agronomy11122521

[ref157] Qi Y. Liu H. Zhang B. Geng M. Cai X. Wang J. . (2022). Investigating the effect of microbial inoculants Frankia F1 on growth-promotion, rhizosphere soil physicochemical properties, and bacterial community of ginseng. Appl. Soil Ecol. 172:104369. doi: 10.1016/j.apsoil.2021.104369

[ref158] Raaijmakers J. M. Mazzola M. (2016). Soil immune responses. Science 352, 1392–1393. doi: 10.1126/science.aaf3252, 27313024

[ref159] Rajani P. Rajasekaran C. Vasanthakumari M. M. Olsson S. B. Ravikanth G. Uma Shaanker R. (2021). Inhibition of plant pathogenic fungi by endophytic Trichoderma spp. through mycoparasitism and volatile organic compounds. Microbiol. Res. 242:126595. doi: 10.1016/j.micres.2020.126595, 33017769

[ref160] Ravanbakhsh M. Kowalchuk G. A. Jousset A. (2019). Root-associated microorganisms reprogram plant life history along the growth-stress resistance tradeoff. The ISME J. 13, 3093–3101. doi: 10.1038/s41396-019-0501-1, 31511619 PMC6863829

[ref161] Raymond N. S. Gómez-Muñoz B. van der Bom F. J. T. Nybroe O. Jensen L. S. Müller-Stöver D. S. . (2021). Phosphate-solubilising microorganisms for improved crop productivity: a critical assessment. New Phytol. 229, 1268–1277. doi: 10.1111/nph.16924, 32929739

[ref162] Reinhold-Hurek B. Bunger W. Burbano C. S. Sabale M. Hurek T. (2015). Roots shaping their microbiome: global hotspots for microbial activity. Annu. Rev. Phytopathol. 53, 403–424. doi: 10.1146/annurev-phyto-082712-102342, 26243728

[ref163] Ribeiro V. P. Gomes E. A. de Sousa S. M. Lana U. G. d. P. Coelho A. M. Marriel I. E. . (2022). Co-inoculation with tropical strains of Azospirillum and Bacillus is more efficient than single inoculation for improving plant growth and nutrient uptake in maize. Arch. Microbiol. 204:143. doi: 10.1007/s00203-022-02759-3, 35044594

[ref164] Roco A. Pérez L. M. (2001). In vitro biocontrol activity of Trichoderma harzianum on *Alternaria alternata* in the presence of growth regulators. Electron. J. Biotechnol. 4, 68–73. doi: 10.2225/vol4-issue2-fulltext-1

[ref165] Rohart F. Gautier B. Singh A. Lê Cao K. A. (2017). mixOmics: An R package for 'omics feature selection and multiple data integration. PLoS Comput. Biol. 13:e1005752. doi: 10.1371/journal.pcbi.1005752, 29099853 PMC5687754

[ref166] Roman-Reyna V. Pinili D. Borjaa F. N. Quibod I. Groen S. C. Mulyaningsih E. S. . (2019). The rice leaf microbiome has a conserved community structure controlled by complex host-microbe interactions. SSRN Elect. J. doi: 10.2139/ssrn.3382544

[ref167] Rosa P. A. L. Mortinho E. S. Jala A. Galindo F. S. Buzetti S. Fernandes G. C. . (2020). Inoculation with growth-promoting Bacteria associated with the reduction of phosphate fertilization in sugarcane Poliana. Front. Environ. Sci. 8:32. doi: 10.3389/fenvs.2020.00032

[ref168] Rosenkranz M. Chen Y. Zhu P. Vlot A. C. (2021). Volatile terpenes–mediators of plant-to-plant communication. Plant J. 108, 617–631. doi: 10.1111/tpj.15453, 34369010

[ref169] Rouphael Y. Franken P. Schneider C. Schwarz D. Giovannetti M. Agnolucci M. . (2015). Arbuscular mycorrhizal fungi act as biostimulants in horticultural crops. Sci. Hortic. 196, 91–108. doi: 10.1016/j.scienta.2015.09.002

[ref170] Rubin R. L. van Groenigen K. J. Hungate B. A. (2017). Plant growth promoting rhizobacteria are more effective under drought: a meta-analysis. Plant Soil 416, 309–323. doi: 10.1007/s11104-017-3199-8

[ref171] Saeed Q. Xiukang W. Haider F. U. Kučerik J. Mumtaz M. Z. Holatko J. . (2021). Rhizosphere bacteria in plant growth promotion, biocontrol, and bioremediation of contaminated sites: a comprehensive review of effects and mechanisms. Int. J. Mol. Sci. 22:10529. doi: 10.3390/ijms221910529, 34638870 PMC8509026

[ref172] Santos L. F. Olivares F. L. (2021). Plant microbiome structure and benefits for sustainable agriculture. Curr. Plant Biol. 26:100198. doi: 10.1016/j.cpb.2021.100198

[ref173] Sasse J. Martinoia E. Northen T. (2017). Feed your friends: do plant exudates shape the root microbiome? Trends Plant Sci. 23, 25–41. doi: 10.1016/j.tplants.2017.09.003, 29050989

[ref174] Schmidt R. Saha M. (2021). Infochemicals in terrestrial plants and seaweed holobionts: current and future trends. New Phytol. 229, 1852–1860. doi: 10.1111/nph.16957, 32984975

[ref175] Schoch C. L. Seifert K. A. Huhndorf S. Robert V. Spouge J. L. Levesque C. A. . (2012). Nuclear ribosomal internal transcribed spacer (ITS) region as a universal DNA barcode marker for Fungi. Proc. Natl. Acad. Sci. 109, 6241–6246. doi: 10.1073/pnas.1117018109, 22454494 PMC3341068

[ref176] Sessitsch A. Pfaffenbichler N. Mitter B. (2019). Microbiome applications from lab to field: facing complexity. Trends Plant Sci. 24, 194–198. doi: 10.1016/j.tplants.2018.12.004, 30670324

[ref177] Shaaban M. Asgari Lajayer B. Nazir G. (2025). Promoting the use of bio-fertilizers to improve soil health. Front. Agron. 7:1730845. doi: 10.3389/fagro.2025.1730845

[ref178] Shade A. Peter H. Allison S. D. Baho D. Berga M. Bürgmann H. . (2012). Fundamentals of microbial community resistance and resilience. Front. Microbiol. 3:417. doi: 10.3389/fmicb.2012.00417, 23267351 PMC3525951

[ref179] Shah A. Nazari M. Antar M. Msimbira L. A. Naamala J. Lyu D. . (2021). PGPR in agriculture: a sustainable approach to increasing climate change resilience. Front. Sustain. Food Syst. 5, 1–22. doi: 10.3389/fsufs.2021.667546

[ref180] Sharifazizi M. Harighi B. Sadeghi A. (2017). Evaluation of biological control of *Erwinia amylovora*, causal agent of fire blight disease of pear by antagonistic bacteria. Biol. Control 104, 28–34. doi: 10.1016/j.biocontrol.2016.10.007

[ref181] Sharpton T. J. (2014). An introduction to the analysis of shotgun metagenomic data. Front. Plant Sci. 5. doi: 10.3389/fpls.2014.00209, 24982662 PMC4059276

[ref182] Shen Z. Ruan Y. Chao X. Zhang J. Li R. Shen Q. (2015). Rhizosphere microbial community manipulated by 2 years of consecutive biofertilizer application associated with banana fusarium wilt disease suppression. Biol. Fertil. Soils 51, 553–562. doi: 10.1007/s00374-015-1002-7

[ref183] Shukla S. K. Shee S. Maity S. K. Solomon S. Awasthi S. K. Gaur A. . (2017). Soil carbon sequestration and crop yields in rice–wheat and sugarcane–ratoon–wheat cropping systems through crop residue management and inoculation of *Trichoderma viride* in subtropical India. Sugar Tech 19, 347–358. doi: 10.1007/s12355-016-0470-x

[ref184] Siegieda D. Panek J. Frąc M. (2023). Plant and soil health in organic strawberry farms–greater importance of fungal trophic modes and networks than α-diversity of the mycobiome. Appl. Soil Ecol. 188:104925. doi: 10.1016/j.apsoil.2023.104925

[ref185] Singh A. Shannon C. P. Gautier B. Rohart F. Vacher M. Tebbutt S. J. . (2019). DIABLO: an integrative approach for identifying key molecular drivers from multi-omics assays. Bioinformatics 35, 3055–3062. doi: 10.1093/bioinformatics/bty1054, 30657866 PMC6735831

[ref186] Specht K. Siebert R. Hartmann I. Freisinger U. B. Sawicka M. Werner A. . (2014). Urban agriculture of the future: an overview of sustainability aspects of food production in and on buildings. Agric. Hum. Values 31, 33–51. doi: 10.1007/s10460-013-9448-4

[ref187] Srivastava P. Singh N. (2021). Effects of microbial inoculants on soil carbon stock, enzymatic activity, and above ground and belowground biomass in marginal lands of northern India. Land Degrad. Dev. 33, 308–323. doi: 10.1002/ldr.4153

[ref188] Sudheer S. Bai R. G. Usmani Z. Sharma M. (2020). Insights on engineered microbes in sustainable agriculture: biotechnological developments and future prospects. Curr. Genomics 21, 321–333. doi: 10.2174/1389202921999200603165934, 33093796 PMC7536804

[ref189] Syed-Ab-Rahman S. F. Carvalhais L. C. Chua E. Xiao Y. Wass T. J. Schenk P. M. (2018). Identification of soil bacterial isolates suppressing different Phytophthora spp. and promoting plant growth. Front. Plant Sci. 9:1502. doi: 10.3389/fpls.2018.01502, 30405657 PMC6201231

[ref190] Tedersoo L. Bahram M. Polme S. Koljalg U. Yorou N. S. Wijesundera R. . (2014). Global diversity and geography of soil fungi. Science 346:1256688–1256688. doi: 10.1126/science.1256688., 25430773

[ref191] Tedersoo L. Tooming-Klunderud A. Anslan S. (2018). PacBio metabarcoding of fungi and other eukaryotes: errors, biases and perspectives. New Phytol. 217, 1370–1385. doi: 10.1111/nph.14776, 28906012

[ref192] Thebault E. Fontaine C. (2010). Stability of ecological communities and the architecture of mutualistic and trophic networks. Science 329, 853–856. doi: 10.1126/science.1188321, 20705861

[ref193] Thilagar G. Bagyaraj D. J. Raoca M. S. (2016). Selected microbial consortia developed for chilly reduces application of chemical fertilizers by 50% under field conditions. Sci. Hortic. 198, 27–35. doi: 10.1016/j.scienta.2015.11.021

[ref194] Thomaier S. Specht K. Hencke D. Dierich A. Siebert R. Freisinger U. B. . (2015). Farming in and on urban buildings: present practice and specific novelties of zero-acreage farming (ZFarming). Renew. Agric. Food Syst. 30, 43–54. doi: 10.1017/S1742170514000143

[ref195] Thompson J. Johansen R. Dunbar J. Munsky B. (2019). Machine learning to predict microbial community functions: An analysis of dissolved organic carbon from litter decomposition. PLoS One 14:e0215502. doi: 10.1371/journal.pone.0215502, 31260460 PMC6602172

[ref196] Tian Y. Liu Y. Yue L. Uwaremwe C. Zhao X. Zhou Q. . (2022). Bacterial inoculant and sucrose amendments improve the growth of *Rheum palmatum* L. by reprograming its metabolite composition and altering its soil microbial community. Int. J. Mol. Sci. 23:1694. doi: 10.3390/ijms23031694, 35163617 PMC8835959

[ref197] Tienda S. Vida C. Lagendijk E. de Weert S. Linares I. González-Fernández J. . (2020). Soil application of a formulated biocontrol rhizobacterium, *Pseudomonas chlororaphis* PCL1606, induces soil suppressiveness by impacting specific microbial communities. Front. Microbiol. 11:1874. doi: 10.3389/fmicb.2020.01874, 32849458 PMC7426498

[ref198] Timmusk S. Behers L. Muthoni J. Muraya A. Aronsson A. C. (2017). Perspectives and challenges of microbial application for crop improvement. Front. Plant Sci. 8:49. doi: 10.3389/fpls.2017.00049, 28232839 PMC5299024

[ref199] Topçuoğlu B. D. Lesniak N. A. Ruffin M. T. Wiens J. Schloss P. D. (2020). A framework for effective application of machine learning to microbiome-based classification problems. MBio 11. doi: 10.1128/mBio.00434-20, 32518182 PMC7373189

[ref200] Toral L. Rodríguez M. Béjar V. Sampedro I. (2020). Crop protection against botrytis cinerea by rhizhosphere biological control agent bacillus velezensis XT1. Microorganisms 8:992. doi: 10.3390/microorganisms8070992, 32635146 PMC7409083

[ref201] Trabelsi D. Ammar H. B. Mengoni A. Mhamdi R. (2012). Appraisal of the crop-rotation effect of rhizobial inoculation on potato cropping systems in relation to soil bacterial communities. Soil Biol. Biochem. 54, 1–6. doi: 10.1016/j.soilbio.2012.05.013

[ref202] Trabelsi D. Mhamdi R. (2013). Microbial inoculants and their impact on soil microbial communities: a review. Biomed. Res. Int. 2013:863240. doi: 10.1155/2013/863240, 23957006 PMC3728534

[ref203] Tripathi B. M. Stegen J. C. Kim M. Dong K. Adams J. M. Lee Y. K. (2018). Soil pH mediates the balance between stochastic and deterministic assembly of bacteria. ISME J. 12, 1072–1083. doi: 10.1038/s41396-018-0082-4, 29515169 PMC5864241

[ref204] Trivedi P. Delgado-Baquerizo M. Trivedi C. Hu H. Anderson I. C. Jeffries T. C. . (2016b). Microbial regulation of the soil carbon cycle: evidence from gene–enzyme relationships. ISME J. 10, 2593–2604. doi: 10.1038/ismej.2016.65, 27168143 PMC5113853

[ref205] Trivedi P. Leach J. E. Tringe S. G. Sa T. Singh B. K. (2020). Plant–microbiome interactions: from community assembly to plant health. Nat. Rev. Microbiol. 18, 607–621. doi: 10.1038/s41579-020-0412-1, 32788714

[ref206] Trivedi P. Trivedi C. Grinyer J. Anderson I. C. Singh B. K. (2016a). Harnessing host-vector microbiome for sustainable plant disease Management of Phloem-Limited Bacteria. Front. Plant Sci. 7. doi: 10.3389/fpls.2016.01423, 27746788 PMC5043059

[ref207] van der Heijden M. G. Hartmann M. (2016). Networking in the plant microbiome. PLoS Biol. 14:e1002378. doi: 10.1371/journal.pbio.1002378, 26871440 PMC4752285

[ref208] van Elsas J. D. Turner S. Bailey M. J. (2003). Horizontal gene transfer in the phytosphere. New Phytol. 157, 525–537. doi: 10.1046/j.1469-8137.2003.00697.x, 33873398

[ref209] Vandenkoornhuyse P. Quaiser A. Duhamel M. Le Van A. Dufresne A. (2015). The importance of the microbiome of the plant holobiont. New Phytol. 206, 1196–1206. doi: 10.1111/nph.13312, 25655016

[ref210] Vannier N. Agler M. Hacquard S. (2019). Microbiota-mediated disease resistance in plants. PLoS Pathog. 15:e1007740. doi: 10.1371/journal.ppat.1007740, 31194849 PMC6564022

[ref211] Velásquez A. C. Castroverde C. D. M. He S. Y. (2018). Plant and pathogen warfare under changing climate conditions. Curr. Biol. 28, R619–R634. doi: 10.1016/j.cub.2018.03.054, 29787730 PMC5967643

[ref212] Vessey J. K. (2003). Plant growth promoting rhizobacteria as biofertilizers. Plant Soil 255, 571–586. doi: 10.1023/A:1026037216893

[ref213] Vink S. N. Chrysargyris A. Tzortzakis N. Salles J. F. (2021a). Bacterial community dynamics varies with soil management and irrigation practices in grapevines (*Vitis vinifera* L.). Appl. Soil Ecol. 158:103807. doi: 10.1016/j.apsoil.2020.103807

[ref214] Vink S. N. Dini-Andreote F. Höfle R. Kicherer A. Salles J. F. (2021b). Interactive effects of scion and rootstock genotypes on the root microbiome of grapevines (Vitis spp. l.). Appl. Sci. 11:1615, 1–11. doi: 10.3390/app11041615

[ref215] Wagner J. Coupland P. Browne H. P. Lawley T. D. Francis S. C. Parkhill J. (2016). Evaluation of PacBio sequencing for full-length bacterial 16S rRNA gene classification. BMC Microbiol. 16:274. doi: 10.1186/s12866-016-0891-4, 27842515 PMC5109829

[ref216] Walters W. A. Jin Z. Youngblut N. Wallace J. G. Sutter J. Zhang W. . (2018). Large-scale replicated field study of maize rhizosphere identifies heritable microbes. Proc. Natl. Acad. Sci. 115, 7368–7373. doi: 10.1073/pnas.1800918115, 29941552 PMC6048482

[ref217] Wang G. Bei S. Li J. Bao X. Zhang J. Schultz P. A. . (2021). Soil microbial legacy drives crop diversity advantage: linking ecological plant–soil feedback with agricultural intercropping. J. Appl. Ecol. 58, 496–506. doi: 10.1111/1365-2664.13802

[ref218] Wang Q. Garrity G. M. Tiedje J. M. Cole J. R. (2007). Naïve Bayesian classifier for rapid assignment of rRNA sequences into the new bacterial taxonomy. Appl. Environ. Microbiol. 73, 5261–5267. doi: 10.1128/AEM.00062-07, 17586664 PMC1950982

[ref219] Wang J. Li R. Zhang H. Wei G. Li Z. (2020). Beneficial bacteria activate nutrients and promote wheat growth under conditions of reduced fertilizer application. BMC Microbiol. 20:38. doi: 10.1186/s12866-020-1708-z, 32085752 PMC7035779

[ref220] Wang Y. Zhao Y. Bollas A. Wang Y. Au K. F. (2021). Nanopore sequencing technology, bioinformatics and applications. Nat. Biotechnol. 39, 1348–1365. doi: 10.1038/s41587-021-01108-x, 34750572 PMC8988251

[ref221] Ważny R. Jędrzejczyk R. J. Rozpądek P. Domka A. Turnau K. (2022). Biotization of highbush blueberry with ericoid mycorrhizal and endophytic fungi improves plant growth and vitality. Appl. Microbiol. Biotechnol. 106, 4775–4786. doi: 10.1007/s00253-022-12019-5, 35729273

[ref222] Ważny R. Rozpądek P. Domka A. Jędrzejczyk R. J. Nosek M. Hubalewska-Mazgaj M. . (2021). The effect of endophytic fungi on growth and nickel accumulation in *Noccaea* hyperaccumulators. Sci. Total Environ. 768:144666. doi: 10.1016/j.scitotenv.2020.144666, 33736318

[ref223] West S. A. Cooper G. A. Ghoul M. B. Griffin A. S. (2021). Ten recent insights for our understanding of cooperation. Nat. Ecol. Evolution. 5, 419–430. doi: 10.1038/s41559-020-01384-x, 33510431 PMC7612052

[ref224] Wilhelm R. C. van Es H. M. Buckley D. H. (2022). Predicting measures of soil health using the microbiome and supervised machine learning. Soil Biol. Biochem. 164:108472. doi: 10.1016/j.soilbio.2021.108472

[ref225] Woo S. L. Ruocco M. Vinale F. Nigro M. Marra R. Lombardi N. . (2014). *Trichoderma*-based products and their widespread use in agriculture. Open Mycol. J. 8, 71–126. doi: 10.2174/1874437001408010071

[ref226] Xu L. Naylor D. Dong Z. Simmons T. Pierroz G. Hixson K. K. . (2018). Drought delays development of the sorghum root microbiome and enriches stress-responsive bacteria. Proc. Natl. Acad. Sci. USA 115, E4284–E4293. doi: 10.1073/pnas.1717308115, 29666229 PMC5939072

[ref227] Yadav S. K. Khokhar U. U. Sharma S. D. Kumar P. (2016). Response of strawberry to organic versus inorganic fertilizers. J. Plant Nutr. 39, 194–203. doi: 10.1080/01904167.2015.1109115

[ref228] Yadav A. N. Kour D. Kaur T. Devi R. Yadav A. Dikilitas M. . (2021). Biodiversity, and biotechnological contribution of beneficial soil microbiomes for nutrient cycling, plant growth improvement and nutrient uptake. Biocatal. Agric. Biotechnol. 33:102009. doi: 10.1016/j.bcab.2021.102009

[ref229] Yang J. Kloepper J. W. Ryu C. M. (2009). Rhizosphere bacteria help plants tolerate abiotic stress. Trends Plant Sci. 14, 1–4. doi: 10.1016/j.tplants.2008.10.004, 19056309

[ref230] Yavitt J. B. Pipes G. T. Olmos E. C. Zhang J. Shapleigh J. P. (2021). Soil organic matter, soil structure, and bacterial community structure in a post-agricultural landscape. Front. Earth Sci. 9, 1–15. doi: 10.3389/feart.2021.590103

[ref231] Yedidia I. Benhamou N. Chet I. (1999). Induction of defense responses in cucumber plants (*Cucumis sativus* L.) by the biocontrol agent Trichoderma harzianum. Appl. Environ. Microbiol. 65, 1061–1070. doi: 10.1007/s00410-001-0323-8, 10049864 PMC91145

[ref232] Yedidia I. Kapulnik Y. Chet I. (2000). Induction and accumulation of PR proteins activity during early stages of root colonization by the mycoparasite *Trichoderma harzianum* strain T-203. Planr Physiol. Biochem. 38, 863–873. doi: 10.1016/S0981-9428(00)01198-0

[ref233] Yilmaz E. Sönmez M. (2017). The role of organic/bio–fertilizer amendment on aggregate stability and organic carbon content in different aggregate scales. Soil Tillage Res. 168, 118–124. doi: 10.1016/j.still.2017.01.003

[ref234] Zancarini A. Westerhuis J. A. Smilde A. K. Bouwmeester H. J. (2021). Integration of omics data to unravel root microbiome recruitment. Cur. Opin. Biotechnol. 70, 255–261. doi: 10.1016/j.copbio.2021.06.016, 34242993

[ref235] Zhao L. Walkowiak S. Fernando W. G. D. (2023). Artificial intelligence: a promising tool in exploring the phytomicrobiome in managing disease and promoting plant health. Plants 12:1852. doi: 10.3390/plants12091852, 37176910 PMC10180744

[ref236] Zhou Y.-H. Gallins P. (2019). A review and tutorial of machine learning methods for microbiome host trait prediction. Front. Genet. 10. doi: 10.3389/fgene.2019.00579, 31293616 PMC6603228

[ref237] Zimina M. Babich O. Prosekov A. Sukhikh S. Ivanova S. Shevchenko M. . (2020). Overview of global trends in classification, methods of preparation and application of bacteriocins. Antibiotics 9, 1–21. doi: 10.3390/antibiotics9090553, 32872235 PMC7559574

